# Oral Primo-Colonizing Bacteria Modulate Inflammation and Gene Expression in Bronchial Epithelial Cells

**DOI:** 10.3390/microorganisms8081094

**Published:** 2020-07-22

**Authors:** Elliot Mathieu, Chad W. MacPherson, Jocelyn Belvis, Olivier Mathieu, Véronique Robert, Vinciane Saint-Criq, Philippe Langella, Thomas A. Tompkins, Muriel Thomas

**Affiliations:** 1 Micalis Institute, AgroParisTech, INRAE, Université Paris-Saclay, 78350 Jouy-en-Josas, France; elliot.mathieu@inrae.fr (E.M.); veronique.robert@inrae.fr (V.R.); vinciane.saint-criq@inrae.fr (V.S.-C.); philippe.langella@inrae.fr (P.L.); 2 Rosell Institute for Microbiome and Probiotics, Lallemand Health Solutions Inc., Montreal, QC H4P 2R2, Canada; cmacpherson@lallemand.com (C.W.M.); jbelvis@lallemand.com (J.B.); omathieu@lallemand.com (O.M.); ttompkins@lallemand.com (T.A.T.)

**Keywords:** oral microbiota, early life, bronchial epithelial cells, immuno-modulation

## Abstract

The microbiota of the mouth disperses into the lungs, and both compartments share similar phyla. Considering the importance of the microbiota in the maturation of the immunity and physiology during the first days of life, we hypothesized that primo-colonizing bacteria of the oral cavity may induce immune responses in bronchial epithelial cells. Herein, we have isolated and characterized 57 strains of the buccal cavity of two human newborns. These strains belong to *Streptococcus*, *Staphylococcus*, *Enterococcus*, *Rothia* and *Pantoea* genera, with *Streptococcus* being the most represented. The strains were co-incubated with a bronchial epithelial cell line (BEAS-2B), and we established their impact on a panel of cytokines/chemokines and global changes in gene expression. The *Staphylococcus* strains, which appeared soon after birth, induced a high production of IL-8, suggesting they can trigger inflammation, whereas the *Streptococcus* strains were less associated with inflammation pathways. The genera *Streptococcus*, *Enterococcus* and *Pantoea* induced differential profiles of cytokine/chemokine/growth factor and set of genes associated with maturation of morphology. Altogether, our results demonstrate that the microorganisms, primo-colonizing the oral cavity, impact immunity and morphology of the lung epithelial cells, with specific effects depending on the phylogeny of the strains.

## 1. Introduction

The buccal cavity (BC) is the body’s entrance for nutriments, air, environmental pollutants and microorganisms. It is a complex ecosystem built by different ecological niches that include the surfaces of hard and soft tissues and saliva [1,2,3]. In humans, the BC is colonized by more than 500 different microbial species, and most of the bacteria belong to the phyla *Firmicutes*, *Proteobacteria*, *Actinobacteria*, *Bacteroidetes* and *Fusobacteria* [1,4,5]. Numerous descriptions have established an association between buccal microbiota and both local and distal diseases [6,7,8]. In respiratory diseases such as asthma, differences in the profile of buccal microbiota have been observed in children as soon as 12 months of age [9]. In addition to modulating the buccal microbiota in other chronic respiratory diseases, the BC may also serve as a potential source of respiratory pathogens [8,10]. The installation of the buccal microbiota matches with the maturation of the lung microbiota; therefore, the exchange of microorganisms (and/or metabolites) between both compartments may impact the course of respiratory diseases. The dense and highly diverse buccal ecosystem, which shares similar phyla with the lung microbiota, is easily accessible to study the characteristics of microorganisms that migrate to the lungs. 

The human lung microbiota has a low density, about 10^3^ bacteria per cm^2^ of lung tissue, and a subtle balance is maintained between microbial immigration and elimination [11,12,13,14,15]. Within this constant balance, the two main bacterial phyla represented are *Bacteroidetes* and *Firmicutes* [12,14,16,17]. It has been shown that the lung microbiota forms within the two to three postnatal months in humans and the first weeks in mice [18,19]. The progressive arrival of these microorganisms coincides with the maturation of the immune system [14]. Increasing evidence suggests that the early lung ecosystem impacts future respiratory health. For example, in mice, specific bacterial stimuli during early life are critical for susceptibility to allergic asthma in young adults [20]. Another example of the importance of early life microbial colonization of the mouse airway is the improved long-term tolerance to allergens through the microbial-induction of a transient peak in expression of the programmed death-ligand 1 (PD-L1) by pulmonary dendritic cells [21]. In humans, lower diversity of microbial exposure in urban than in rural areas results in a higher incidence of allergy and asthma [22,23,24]. These observations, among others, support the hypothesis that exposure to a wide range of diverse microbial signals during the first few months of life has a major impact on the susceptibility to respiratory diseases such as asthma [25,26,27,28]. The postnatal period is a pivotal time in respiratory health, making the primo-colonizing bacteria of special interest to screen for new probiotics.

Cells that compose the airway epithelium act as the first line of defense against the external environment and are able to initiate innate and adaptive immune responses [29,30,31]. Bronchial epithelial cells (BEC) express various innate sensors (e.g., toll-like receptors (TLRs), NOD-like receptors (NLRs) and C-type lectins receptors (CLRs)) that can detect microbes and activate molecular cascades in host cells, triggering the induction of tolerance or inflammation [32,33]. The mediation and regulation of the immune responses are controlled by the secretion of cytokines, chemokines and growth factors. Cytokine secretion, for example, is a major feature of the inflammatory process in allergic reaction. In asthma, changes in the epithelium structure, disruption of epithelial tight junctions and epithelium thickening are observed [30,34]. The physiological characteristics of the bronchial epithelium, which are modulated during disease, influence the homeostasis between the lung and its microbiota [14]. For example, a longer residence time of mucus in the airways may favor the selection of certain bacteria with a high tropism for mucus, leading to the persistence of pathogens [35]. These observations highlight the importance of the BECs in respiratory health and disease, making them a target of importance to study the relationship between external signals and the host.

We hypothesized that early life buccal bacteria induce specific immunomodulatory responses in BECs. The goal of our study was to isolate commensal bacterial strains from the BC of babies and to elucidate their impacts on the immune modulation and global changes in gene expression in BECs. The effect of *Streptococcus*, *Enterococcus* and *Pantoea* strains on BEAS-2B cells was evaluated by using genome-wide human expression microarrays and cytokine and chemokine multiplexing immunoassays. The isolation of buccal commensals and the understanding of their interaction with the BECs is a first step toward understanding the underlying cellular mechanisms at play, the identification of novel beneficial microbes and the possible management of respiratory health.

## 2. Materials and Methods 

### 2.1. Bacterial Strains, Media and Growth Conditions

Bacterial strains were isolated from the BC of human newborns, using swabs (Portagerm Amies agar and swab—sterile zone, Biomerieux, Marcy-l'Étoile, France). Donors were two males born via vaginal route at term, and the mothers did not receive antibiotic therapy during pregnancy. After written consent was obtained, a pack of 10 swabs was given to the parents. Sampling was performed by the parents as soon as the first day of life, once or twice daily and for 5 to 10 consecutive days. Samples were stored at 4 °C until collection. Swabs were placed in sterile Brain Heart Infusion (BHi) (BD/Difco, Fisher Scientific, Thermo Fisher Scientific, Waltham, MA, USA) broth medium supplemented with 5 g·L^−1^ of yeast extract (Gibco^®^, Thermo Fisher Scientific, Waltham, MA, USA). After dilution, bacteria were cultivated on BHi agar medium supplemented with 10 mL·L^−1^ of hemin (H 9039, 50 mL/100 mL) and 5 g·L^−1^ of yeast extract. Plates were incubated for 24–48 h, at 37 °C, under aerobic and anaerobic conditions. Representative bacterial colonies were selected based on the difference in shape, size and color. Isolated strains were subcultured on BHi agar medium, and the purity of the culture was determined before storage in 16% glycerol at −80 °C.

### 2.2. Bacterial Strain Identification

Strains were identified at the genus level, using sequencing PCR amplicons of 16S rRNA genes. 16S PCR was performed by using the primers 1492R (5′-ACGGCTACCTTGTTACGACTT-3′, position 1517R) and 27F (5′-AGAGTTTGATCCTGGCTCAG-3′, position 008F) [36]. PCR-amplified sequences were run on 1 % agarose gel and then sent for sequencing to Eurofins (Eurofins Scientific, Germany). Nucleotide sequences were analyzed by using Basic Local Alignment Search Tool (BLAST) and compared to the NCBI non-redundant database [37].

### 2.3. Bacterial Strains Preparation for Challenge Assay

Bacterial strains were thawed and then incubated in BHi. After overnight culture, cultures were passaged once, and on the day of the challenge assay, bacterial cultures were centrifuged, and the pellet was washed with 1 mL of phosphate buffered saline (PBS) (Gibco^®^, Thermo Fisher Scientific, Waltham, MA, USA). Bacterial cultures were used in post-logarithmic phase, and therefore the time of culture was adapted for each strain. After a second centrifugation, PBS was removed, and bacterial pellet was resuspended in Roswell Park Memorial Institute (RPMI) 1640 Medium (Lonza, Basel, Switzerland). Bacterial cells were enumerated by using the BD Accuri™ C6 (BD Biosciences, Franklin Lakes, NJ, USA) and stored on ice until further used, for 10 min.

### 2.4. BEAS-2B Human Bronchial Epithelial Cell Challenge

BEAS-2B human bronchial epithelial cell line was obtained from the American Type Culture Collection (ATCC CLR-9609). Cells were maintained in RPMI-1640 medium supplemented with 10% fetal bovine serum (Eurobio, Les Ulis, France), 1% penicillin/streptomycin (Sigma-Aldrich, St. Louis, MO, USA) and 1% L-glutamine (Gibco^®^, Thermo Fisher Scientific, Waltham, MA, USA) in 75 cm^2^ tissue culture flask (Sarstedt, Nümbrecht, Germany) and incubated at 37 °C, in a 5% CO2 atmosphere, in a humidified incubator. Cells were passaged before reaching 80% confluency, using Tripsin-EDTA (Gibco^®^, Thermo Fisher Scientific, Waltham, MA, USA). To prepare the challenge assay, cells were detached and enumerated, using an automated cell counter (Bio-Rad TC20 (Bio-Rad, Hercules, CA, USA), and seeded at 5 × 10^4^ cells/mL, in a 25 cm^2^ tissue culture flask (TPP, Trasadingen, Switzerland) (T25) with 5 mL fresh medium, and incubated at 37 °C, 5 % CO2. At 80% confluency, cells were challenged with the bacterial treatments. Prior to the challenge, BEAS-2B cells were enumerated, and appropriate bacterial concentration was calculated in order to get a MOI of 100:1 (100 bacterial cells for each BEAS-2B cell). Medium was removed from the T25, and 5 mL of one of the bacterial conditions, diluted in RPMI-1640 (without supplements), was added to the flask. After a 6 h incubation period at 37 °C, 5 % CO2, supernatants were collected, and Bovine Serum Albumin (BSA) (Sigma-Aldrich, St. Louis, MO, USA), at a final concentration of 0.5 %, was added to each sample, to prevent protein degradation. Supernatants were then stored at −80 °C, and RNA was extracted from the challenged cells.

A similar protocol, in which 24-well plates replaced T25, was used to generate IL-8 secretion data.

### 2.5. Human Cytokine and Chemokine Profiling

Cytokine and chemokine profiling was done by using the Bio-Plex Pro™ 27-Plex Human Cytokine Panel (Bio-Rad, Hercules, CA, USA), the Bio-Plex Pro™ 40-Plex Human Chemokine Panel (Bio-Rad, Hercules, CA, USA) and the single-Plex TSLP (Bio-Rad, Hercules, CA, USA). For all plexes, all cytokines and chemokines were multiplexed on the same 96-well plate (1 plate for each plex). Cytokine and chemokine standards were serially diluted, and protein profiling from all challenges were done as per the manufacturer’s instructions (Bio-Rad), with 4 biological replicates. Quality controls (from the kit) were also included, only for the 40-plex, to ensure the validity of the results obtained. Protein concentrations were calculated by using the Bio-Plex ManagerTM software and expressed in pg/mL. 

IL-8 was quantified by using ELISA Max™ Standard Set Human IL-8 (BioLegend, San Diego, CA, USA); manufacturer instructions were followed.

### 2.6. RNA Extraction and RNA Cleanup

After the challenge assay, supernatant was removed, and 2 mL Trizol Reagent (Invitrogen, Carlsbad, CA, USA) was added to the 25 cm^2^ tissue culture flask. After 5 min incubation with gentle agitation at room temperature, cell lysates were transferred to two PhaseLock tubes (Quantabio, Beverly, MA, USA), and 0.2 volumes of chloroform (*v/v*) was added to each tube. Tubes were shacked vigorously and centrifuged (12,000× *g* for 10 min at 4 °C). Aqueous phase containing RNA was transferred to a new tube, and RNA was precipitated by adding 0.5 volumes (*v/v*) of isopropyl alcohol. After centrifugation (12,000× *g* for 10 min at 4 °C), RNA pellets were washed with 70% ethanol and dried by placing them into a flow hood. Pellets were resuspended in 20 µL RNase-free water, and a step of RNA cleanup was performed, following the manufacturer’s instructions (RNeasy mini kit, Qiagen, Hilden, Germany). RNA was quantified by using Nanodrop, and RNA integrity (RIN) was determined with Bioanalyzer (RNA 6000 Nano Kit, Agilent Technologies, Santa Clara, CA, USA). RNA was then stored at −20 °C, until needed. Only RNA samples with a RIN > 8 were used in reverse transcription (RT) of mRNA into cDNA for subsequent gene expression microarrays [38].

### 2.7. RNA Reverse Transcription and Fluorescent Labeling

First, 10 µg aliquots of RNA were SpeedVac. As previously described by MacPherson et al., 10 µL of freshly prepared oligo dT-master mix (1.5 µL of oligo dT23 primers (3 µg/µL) and 8.5 µL of RNase free water per sample) were added to samples and then incubated at 70 °C for 10 min, in a dry bath [38]. Two cDNA synthesis master mixes were prepared by mixing 4 µL of 5X First Strand Buffer, 2 µL of 0.1 M DTT, 2 µL of dNTP home mix (6.67 mM of GTPs, dATPs, dTTPs and 2 mM of dCTPs), 1 µL of Superscript III (200 U/µL) and 1 µL of a 1 mM mix of Cy3 or Cy5, for each sample. Then, 10 µL of freshly prepared cDNA master mix was added per sample. After gentle flicking, tubes were incubated at 42 °C for 3 h, in a dry bath protected from light. After incubation, 1 µL of RNase mix (0.05 mg/mL RNase A and 0.05 U/µL RNase H) was added to each sample and incubated at 37 °C for 30 min. Labeled cDNA was purified, following the manufacturer’s instructions, to remove unincorporated Cy3 or Cy5 dyes (PCR Purification Kit, Qiagen, Hilden, Germany). Labeled and purified cDNA samples were stored with a foil cover at −20 °C, until needed.

### 2.8. Microarrays Analysis

Genome-wide expression analysis was performed by using Agilent Whole Human 4x44K microarrays (Agilent Technologies, Santa Clara, CA, USA). Microarrays were prepared for pre-hybridization, hybridization and post-hybridization, as previously described MacPherson et al. [38]. Arrays were scanned at 10 μm resolution, using a ScanArray 5000 instrument from Perkin-Elmer (Waltham, MA, USA) and ScanArray software (version 3.0). Images for Cyanine 5 and Cyanine 3 were saved as TIFF format, and a composite image was created and saved as JPEG. 

### 2.9. LDH Cytotoxicity Assay

LDH was quantified by colorimetric method in the supernatant of cell culture by using the Pierce LDH Cytotoxicity Assay Kit (Thermo Fisher Scientific, Waltham, MA, USA). BEAS-2B Bronchial epithelial cells were challenged with bacterial strains, as described in Section 2.4, BEAS-2B Human Bronchial Epithelial Cell Challenge. After 6 h of incubation, cell supernatants were recovered, and LDH was quantified, following the manufacturer’s instructions. Briefly, 50 µL of supernatants was transferred to a 96 wells-plate, and 50 µL of the reaction mixture (containing Substrate mix and Assay buffer) was added to each well. After 30 min incubation at room temperature, 50 µL of Stop solution was added into each well. To determine LDH activity, absorbance at 490 and 680 nm was measured by using a plate-reading spectrophotometer. To obtain the maximum LDH activity control (“Lysis 3 h” in Figure S2), BEAS-2B cells were challenged for 3 h at 37 °C, 5 % CO2 with Lysis buffer (10 % in RPMI). Spontaneous LDH activity control corresponds to RPMI medium only. The percentage of cytotoxicity was calculated as follows:

(1) $$\%\;Cytotoxicity=\frac{Bacteria\;treated\;LDH\;activity-Spontaneous\;LDH\;activity}{Maximum\;LDH\;activity-Spontaneous\;LDH\;activity}\times 100$$

### 2.10. Data and Statistical Analysis

#### 2.10.1. Human Cytokine and Chemokine Profiling 

All data are shown as the mean pg/mL ± standard error of the mean (SEM). Statistical analysis was performed by using GraphPad Prism’s Version 8 (GraphPad Software, Inc., San Diego, CA, USA), one-way analysis of variance (ANOVA) or non-parametric Kruskal–Wallis test followed by Dunn’s multiple comparison test were used to determine statistical significance. The *p*-values are as follows: *: *p* < 0.03, **: *p* < 0.002, ***: *p* < 0.0002 and ****: *p* < 0.0001. 

For each condition and cytokine/chemokine, ratios were calculated as follows:

(2) $$Ratio=\frac{\left(bacteria\;stimulated\;average\right)}{\left(medium\;average\;\right)}$$

*Ratio* values were then entered into MeV to create the heat map and hierarchical clustering gene leaf order; Euclidian distance was used as distance metric.

#### 2.10.2. Microarray Data Processing

The signal intensity of all spots was quantified and normalized (Global LOWESS) by using ImaGene version 9.0 (BioDiscovery, El Segundo, CA, USA). Statistical analysis was done with MultiExperiment Viewer (MeV) version 4.8 (TM4 microarray software suite, J. Craig Venter Institute, Rockville, MD, USA). Genes were considered significantly differentially expressed when (a) a *t*-test *p*-value of less than 0.05 and (b) a cutoff in transcript abundance of least 1.5-fold change were reached. 

Ingenuity Pathway System (IPA) was used to discover relevant biological patterns and genes network modulated by the bacterial challenges. IPA is a web-based bioinformatics application for analyzing and understanding large gene-expression datasets. A *p*-value < 0.05 (-log(*p*-value) > 1.3) was considered to be statistically significant for the enrichment of pathways in IPA (*p-*value calculated by using Fisher’s exact test).

Information about the microarray platform and the expression data files can be found on the NCBI Gene Expression Omnibus (GEO; http://www.ncbi.nlm.nih.gov/geo/) under GEO Series record GSE154245.

## 3. Results

### 3.1. Strains Isolated from the Swabs

**Figure 1:**
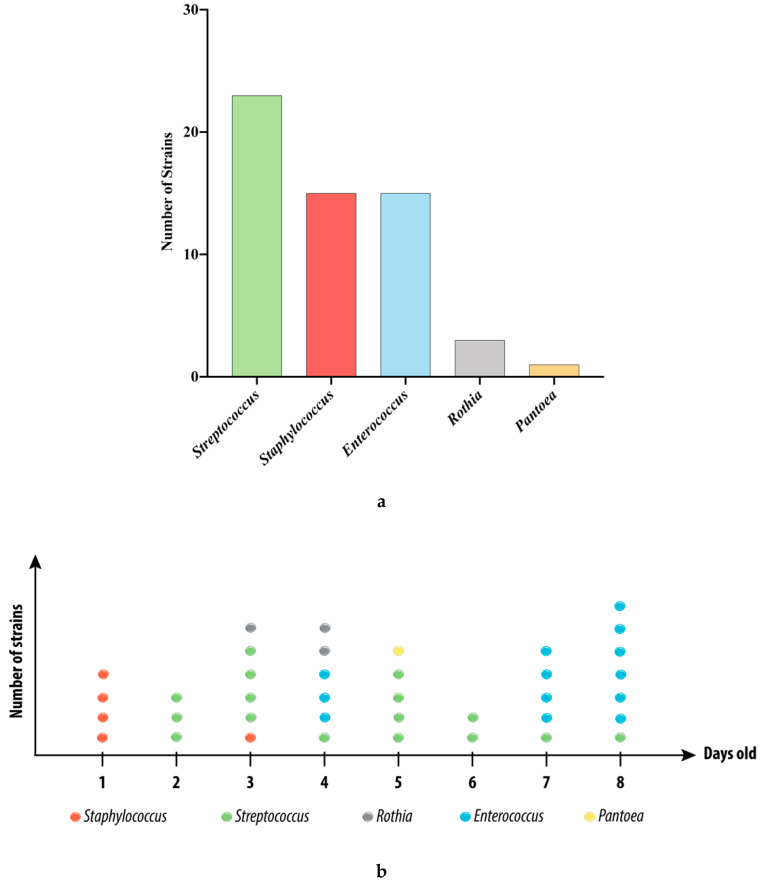
The bacterial library contains members of the early life buccal core microbiota. (**a**) Genera repartition of the 57 strains identified in all the samples (days are mixed) from both donors. (**b**) Bacterial distribution through the days for one donor (*n* = 38). Strains were identified at the genus level, using 16S PCR sequencing. Data are presented as the number of strains per genera. Each dot represents a strain.

A total of 57 isolates were isolated from the BC of two babies (males, born by vaginal route) and identified by using 16S PCR sequencing. When assessing the abundance in the samples from both donors, the genus *Streptococcus* was the most represented, with 23 members (Figure 1a). *Staphylococcus* and *Enterococcus* strains (*n* = 15 each) were in a close amount. Three sequences were identified as strains belonging to the genus *Rothia* and one to the genus *Pantoea*. We next displayed the distribution of the strains over eight days for one donor (Figure 1b). Note that we were not able to perform such kinetics for the second donor (due to the lack of sampling or the difficulty in purifying strains), making the comparison between the two donors impossible. As shown in Figure 1b, the *Staphylococcus* strains were mainly isolated from the BC samples from the first day of life and were rapidly replaced by the other genera. The *Streptococcus* strains were isolated from day two and were present in all the samples from this day. The three *Rothia* strains of the library were isolated from this donor on days three and four. The *Pantoea* strain was isolated on day five, and, finally, the *Enterococcus* strains were the most isolated in the samples on days seven and eight.

### 3.2. Cytokines and Chemokines Production Profiling in BEAS-2B Cells, Following Bacterial Treatments

**Figure 2:**
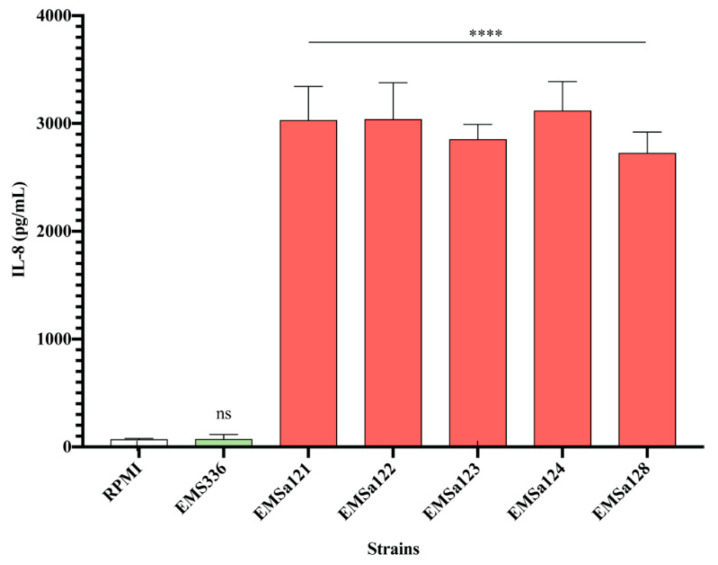
IL-8 produced by the BEAS-2B cells in response to primo-colonizing *Staphylococcus* strains. All strains of *Staphylococcus* (EMSa) and *Streptococcus* (EMS) were isolated from the buccal cavity of human newborns. *Staphylococcus* was isolated on the first day of life. For the challenge assay, BEAS-2B cells were co-incubated with bacterial strains (MOI of 100:1) for 6 h in 24-well plates. IL-8 was quantified by using ELISA immunoassays. Data are the mean of secreted IL-8 (pg/mL) ± SEM of at least two biological replicates (with two technical replicates each). *p*-values: *: *p* < 0.03, **: *p* < 0.002, ***: *p* < 0.0002 and ****: *p* < 0.0001.

**Figure 3:**
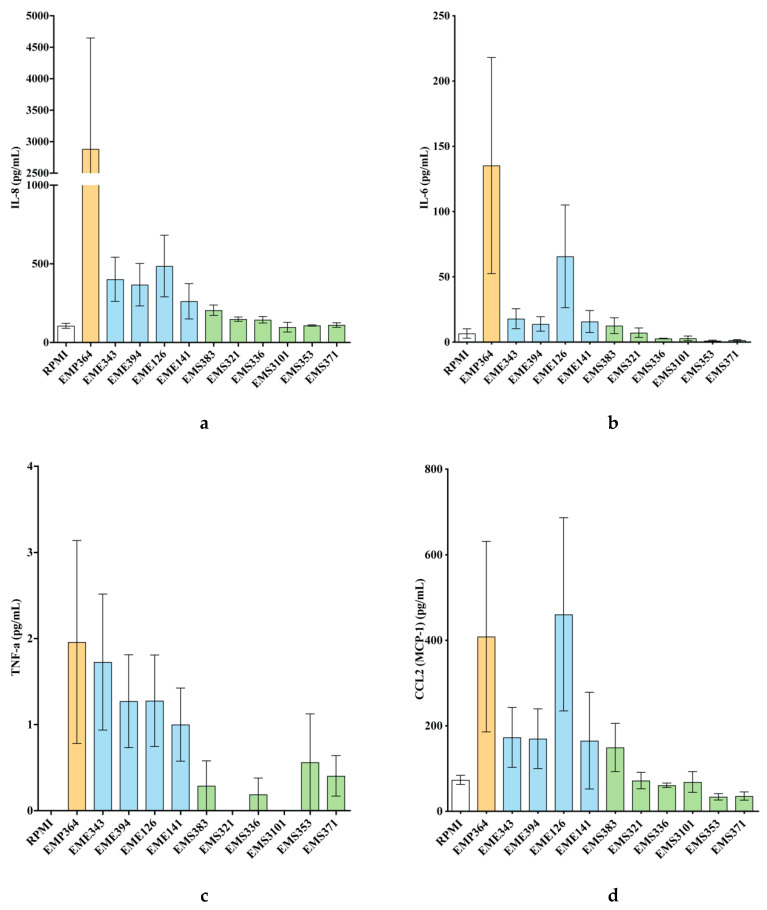

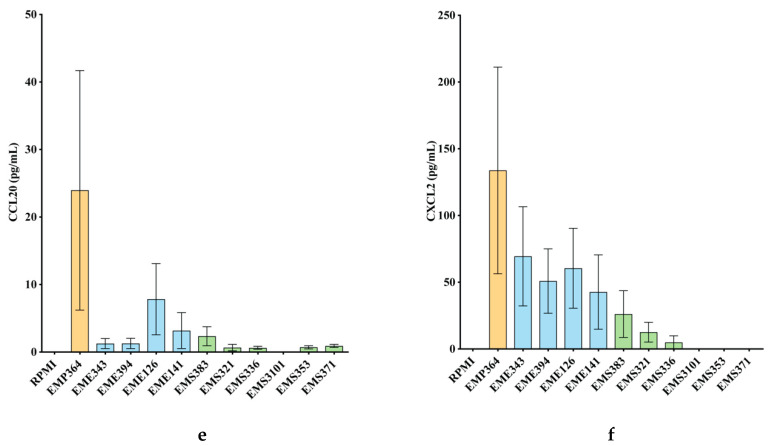
Cytokines and chemokines profiling of the BEAS-2B cells in response to bacterial stimulation: (**a**) IL-8, (**b**) IL-6, (**c**) TNF-α, (**d**) CCL2, (**e**) CCL20 and (**f**) CXCL2. Strains are identified as follows: *EMS*, *Streptococcus* strains; *EME*, *Enterococcus* strains; *EMP*, *Pantoea*. For the challenge assay, BEAS-2B cells were co-incubated with bacterial strains (MOI of 100:1) for 6 h, in 25 cm^2^ tissue culture flasks. Cytokines and chemokines were quantified by using multiplex-magnetic-beads-based assay. Data are the mean of secreted proteins (pg/mL) ± SEM of four biological replicates (with two technical replicates each). *p*-values: *: *p* < 0.03, **: *p* < 0.002, ***: *p* < 0.0002 and ****: *p* < 0.0001.

**Figure 4:**
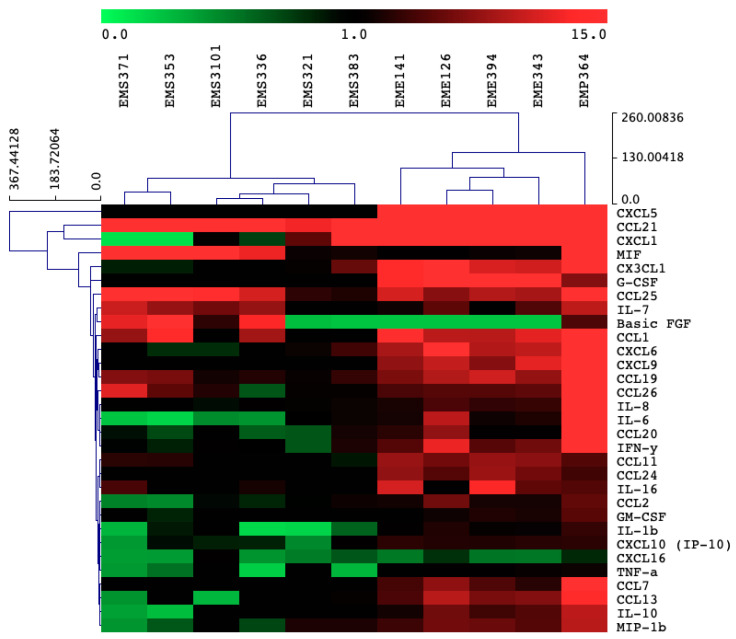
Similar patterns of cytokine, chemokine and growth factor production can be observed depending on the genera. Heatmap showing ratios (treatment/control, values not shown) of secreted proteins after 6 h incubation with bacterial strains. Strains are identified as follows: *EMS*, *Streptococcus* strains; *EME*, *Enterococcus* strains; *EMP*, *Pantoea*. Data are the mean of the ratio of four biological replicates. Heat map produced by using MeV. Hierarchical clustering gene leaf order, and Euclidian distance used as distance metric.

From these 57 strains, we selected 11 strains of interest, which comprised *Streptococcus*, *Enterococcus* and *Pantoea*. The other strains (46 strains) mainly displayed a pro-inflammatory profile after a co-incubation with the BEAS-2B cells (data not shown). Particularly, the *Staphylococcus* strains isolated from the first day of life induced Interleukin (IL)-8 secretion (Figure 2). We evaluated the impact of the 11 bacterial strains on the protein levels of cytokine and chemokine secreted by the BEAS-2B cells in the supernatant, following challenge with the bacterial strains. Cytokines and chemokines were quantified by using fluorescent-magnetic-bead-based multiplex immunoassay. Most of our bacterial treatments did not significantly increase of pro-inflammatory markers such as IL-8, IL-6, TNFα (Figure 3a–c) and IL-1β (Figure S1) in comparison to controls. Only the *Pantoea EMP364* strain induced significant production of these pro-inflammatory proteins (Figure 3a–c). Most strains did not induce more than 10.1 % cytotoxicity (measured by LDH release) (Figure S2), although *Streptococcus EMS383* induced up to 60% cytotoxicity. None of the conditions induced Th2-related cytokines IL-2, IL-4, IL-5, IL-7, IL-9, IL-12p70, IL-13 and IL-17A secretion by the BEAS-2B cells following bacterial treatments (data not shown). *Pantoea EM-364* produced an increased production of chemokine markers such as C-C Motif Chemokine Ligand (CCL) 20 and C-X-C Motif Chemokine Ligand (CXCL) 2 (Figure 3e,f). Although not significant, the *Enterococcus* strains increased the production of CCL2 and CXCL2 (Figure 3d,f). Along with *Pantoea EM-364*, they also induced the production of most of the chemokines measured (Figure S1). *Streptococcus* strains only increased CCL25 secretion by the BEAS-2B cells (Figure S1). Most of the *Streptococcus* strains promoted the production of FGF basic (Figure S1), e.g., *EMS353*: 89.80 pg/mL ± 16.19 vs. RPMI: 4.37 pg/mL ± 2.12), while the *Enterococcus* strain had no effect on the production of this growth factor. The opposite pattern was observed for granulocyte colony-stimulating factor (G-CSF) release (Figure S1; e.g., *EME343*: 29.25 pg/mL ± 4.51 vs. RPMI: 0 pg/mL ± 0). The macrophage migration inhibitory factor (MIF) was strongly secreted by BEAS-2B cells in response to *Streptococcus EMS336* (19,847 pg/mL ± 9844), EMS3101 (40,117 pg/mL ± 20,635), *EMS353* (73,039 pg/mL ± 21,501) and *EMS371* (124,956 pg/mL ± 47,693), while all the *Enterococcus* strains had no effect on the production of this cytokine (Figure S1). Figure 4 shows the heat map of the ratios for each cytokine and chemokine detected in each condition and demonstrates that cytokine and chemokine production, by the BEAS-2B cells, was genus-specific. Indeed, similar secretion patterns within the *Streptococcus* genus were seen, as well as within the *Enterococcus* genus.

### 3.3. Analysis of Differential Gene Expression of BEAS-2B Cells, Using Microarray Analysis

**Figure 5:**
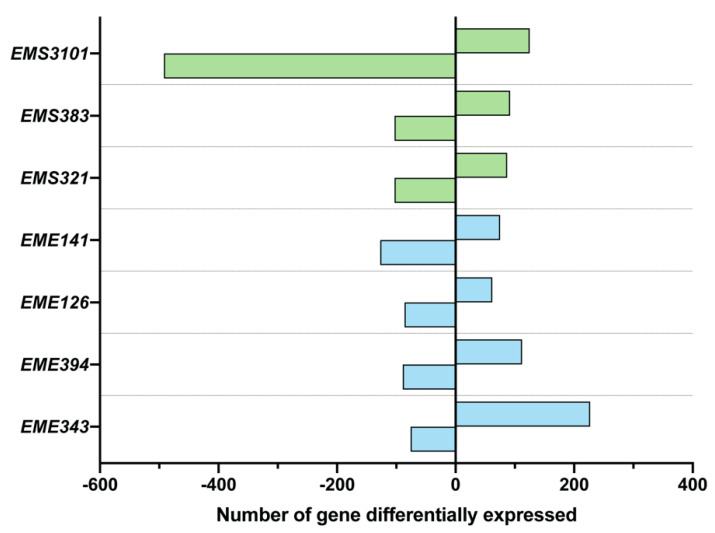
The number of differentially expressed genes is specific to the strain. Following bacterial challenge for 6 h, RNA from the BEAS-2B cells culture was analyzed by genome-wide expression analysis, using Agilent Whole Human 4x44K microarrays. Gene expression changes were expressed as fold of bacteria-treated compared to untreated control (culture medium only). Genes were considered significantly differentially expressed when (i) a t-test *p-*value of less than 0.05 and (ii) a cutoff in transcript abundance of least 1.5-fold change were observed. Data are expressed as the number of gene differentially expressed. Strains are identified as follows: *EMS*, *Streptococcus* strains; *EME*, *Enterococcus* strains.

To determine if early life buccal commensal bacteria influenced the transcriptional response of BECs, Human bronchial epithelial BEAS-2B cells were challenged with bacterial cultures (MOI = 100) for 6 h. RNA was extracted, and cDNA was prepared for a comparative microarray analysis of BEAS-2B cells stimulated individually with a selection of bacterial strains, or with quiescent cells serving as the control. Note that we were able to recover RNA from the flasks of 7 out of the 11 bacterial co-culture. As shown in Figure 5, the number of genes differentially expressed was strain-dependent. Most of the bacterial conditions differentially regulated between 148 and 303 genes; however, *Streptococcus EMS3101* downregulated up to 617 genes. This last treatment modulated a high number of genes (Figure 5). Among the whole set of genes, *IL-8*, *CCL2* and Activating Transcription Factor 3 (*ATF3*) were strongly upregulated (Table 1), and IL-8 was the most differentially expressed in BEAS-2B co-cultured with three out of four *Enterococcus* strains, with a maximum fold change of 14.5 for *EME343*.

### 3.4. Gene Enrichment Analysis

**Figure 6:**
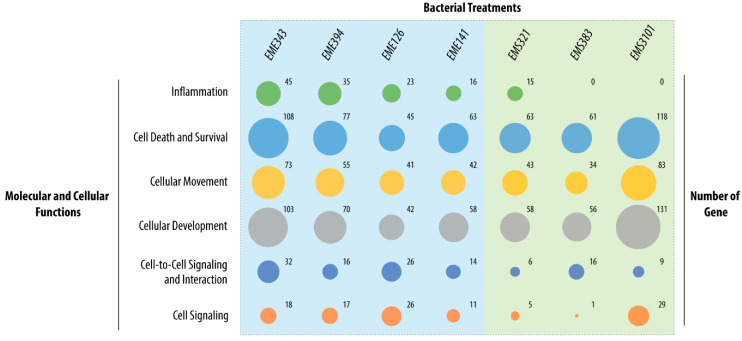
Number of genes significantly enriched in top molecular and cellular functions. Analysis was performed by using IPA. The area of the bubbles is proportional to the number of genes differentially expressed. Enrichment analysis of molecular and cellular functions are statistically significant when a *p*-value <0.05. The *p*-values range from 1.15 × 10^−2^ to 1.29 × 10^−13^.

In order to identify relevant biological patterns from the whole set of genes differentially regulated by the BC strains, enrichment analysis was performed by using Ingenuity Pathway Analysis (IPA) software. The enrichment and pathway analysis was realized on gene sets with a 1.5-fold-change cutoff for each condition. The analysis revealed enrichment in molecular and cellular functions corresponding to the gene sets related to “cell signaling”, “cell death and survival”, “cellular movement”, “cellular development”, “cell-to-cell signaling and interactions” and “inflammation”. As shown in Figure 6, the different bacteria induced strain-specific gene modulation in these clusters of molecular and cellular functions. Overall, *Streptococcus EMS3101* and *Enterococcus EME343* were the two strains that induced the most gene modulation in the assay conditions used. For example, the *Streptococcus EMS3101* had the greatest impact on genes involved in “cell death and survival”, “cellular movement” and “cellular development” related functions. Interestingly, for the *Streptococcus*, only *EMS321* was found to regulate genes enriched in the “inflammation” cluster. All the *Enterococcus* strains displayed a similar pattern of enrichment. Of note, the *EME141* strain had less impact on genes differentially expressed related to “inflammation”, “cell-to-cell signaling” and “interactions and cell signaling” functions than the other *Enterococcus* strains. 

**Figure 7:**
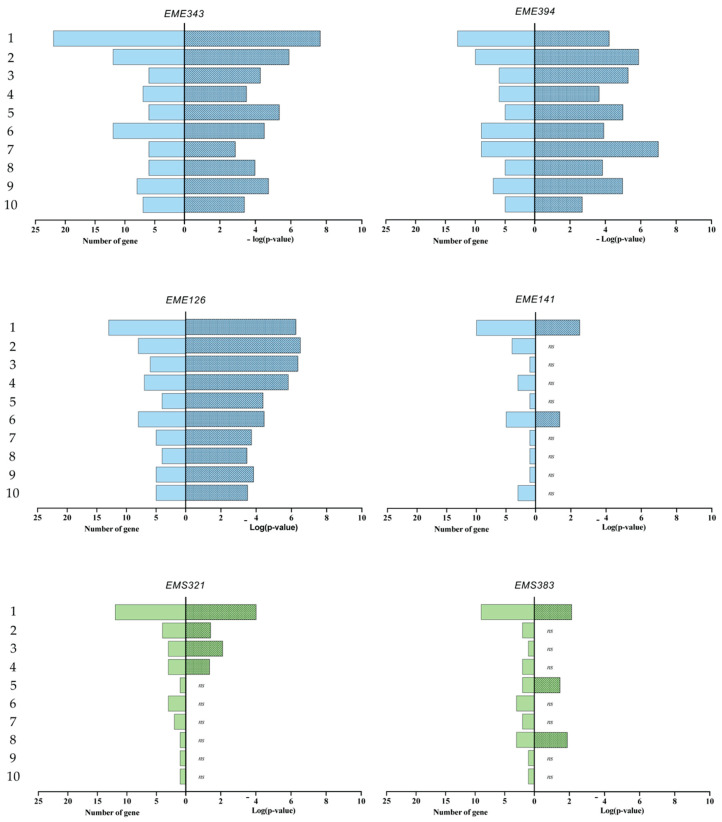

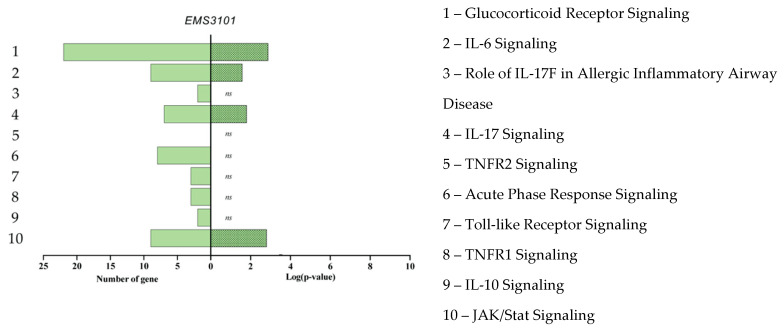
Significant canonical pathways of inflammation and the immune system modulated in BEAS-2B cells by our BC bacteria. Strains are identified as follows: *EMS*, *Streptococcus* strains; *EME*, *Enterococcus* strains. Fold change cutoff = 1.5. The *y*-axis is the −log(*p-*value), as produced by IPA, and the *x*-axis is the pathways modulated in BEAS-2B cells. The canonical pathways are predicted pathways that are changing based on gene expression; the higher the −log(*p-*value) is, the less likely the association is random.

To get further insight into the pathways impacted by the bacterial treatments, IPA canonical pathway analysis was performed. We mainly focused our attention to pathways related to the “inflammation”, “cell-to-cell signaling and interactions” and “cell signaling functions” to assess the immunomodulatory impact of BC strains on airway epithelial cell immunity. Based on IPA, enrichment pathways of differentially expressed genes were observed for glucocorticoid receptor signaling, IL-6 signaling, role of IL-17F in allergic inflammatory airway diseases, IL-17 signaling, tumor necrosis factor receptor (TNFR) 1 and 2 signaling, acute phase response signaling, TLRs signaling, IL-10 signaling, and the Janus kinase/signal transducers and activators of transcription (JAK/STAT) signaling (Figure 7). Overall, this analysis confirmed that *Enterococcus* strains had the strongest impact on the selected pathway of the inflammation and cell signaling. However, the EME141 strain had a significant lower impact on these pathways compared to the other *Enterococcus* strains. Only the glucocorticoid receptor signaling and the acute phase response signaling pathways were significantly enriched from the gene set corresponding to EME141. The *Streptococcus* strains were significantly associated in less than half of the selected pathways, and these associations were more likely to be random than with the *Enterococcus* strains (except for EME141) (Figure 7). Examples of genes that were upregulated in these pathways included *IL-8* (14.5-fold), *CCL2* (9.67-fold), *ATF3* (7.88-fold), *CXCL2* (7.99-fold) and *IL-1A* (up to 9.57-fold). 

## 4. Discussion

This study aimed to isolate cultivable commensal strains from the buccal cavity of human newborns and evaluate their impact on bronchial epithelial cells. We isolated pioneer members of the early life buccal microbiota, and our results provide new insight on the ability of primo-colonizing bacteria to modulate inflammatory pathways in BEAS-2B cells. We also showed that strains from the different genera induced specific differential cytokine/chemokine/growth factor secretion profiles. All together, these data suggest that early life buccal bacterial strains may differentially stimulate and shape the lung immunity. 

The isolation process led to the creation of a library made by bacterial strains originating from the oral cavity of human newborns. It is important to note here that the methods of sampling, storage, transport and isolation used in this study may have resulted in the loss of strict anaerobic, sub-dominant and fastidious bacteria. However, the objective of this study was not to identify all the cultivable bacteria of the BC; instead, we sought some representative strains, in order to assess their immunomodulatory effects. Strains that were identified belong to genera that have been shown to be part of the buccal core microbiota during early life. A recent study by Daspher and colleges has shown that members of *Streptococcus*, *Staphylococcus* and *Rothia* genera were early life colonizers of the BC [39]. In our library, *Enterococcus* species were also present. This genus is generally less represented in buccal and lung microbiome [40]. Based on the strains we have identified, the colonization of the BC appears to be sequential. *Staphylococcus* was the only genus detected on the first day of life, while, apart from being the most isolated genus, the *Streptococcus* strains were isolated in every sample from day two onward (Figure 1b), suggesting a persistent colonization of the oral cavity by this genus rather than a transitory passage. Our findings are in accordance with previous studies that have shown that *Streptococcus* is the dominant genus in the oral cavity of newborns [41,42,43,44]. Our intention to target the primo-colonizing bacteria was motivated by recent reports describing the early life as a pivotal time for determining the susceptibility to develop chronic respiratory diseases such as asthma. In particular, Arrieta and colleagues have suggested that the first 100 days of life represent an “early-life critical window” where gut dysbiosis influences the risk to develop asthma [45]. The authors demonstrated a causal role of certain bacterial taxa (e.g., *Veillonella* and *Faecalibacterium*) during this period in averting asthma development. Furthermore, increasing evidence suggests that the early lung ecosystem impacts the future respiratory health [20,21]. While no causal role has been established for the microbial communities of the BC in asthma, a recent report has highlighted changes in the composition of the buccal microbiota as soon as three months of age in infants that further developed allergy and asthma later in life [9]. Herein, we had access to precious early life microbial strains of the BC that may be an important stimulator of local and distal immunity. The characterization of their immunomodulatory potential is of major interest in the development of new therapeutic approach for the management of chronic respiratory diseases. 

An important observation in this study is that primo-colonizing bacteria clearly induced different response profiles, depending on their phylogeny. Interestingly, the *Staphylococcus* strains isolated in the first days of life displayed strong pro-inflammatory profiles. As shown in Figure 2, some strains were able to induce high production of IL-8. We can presume that there is a mutual benefit for the host and for the *Staphylococcus* strains to this pro-inflammatory response. Thus, it would be interesting to determine the impact of these pro-inflammatory stimuli at birth, which may participate in the acquisition of tolerance by the innate system. We focused our attention on a selection of 11 strains and the measurement of cytokines and chemokines in BEAS-2B cell supernatants. Our results have shown that the *Streptococcus* and *Enterococcus* strains were inducing differential production profiles. Overall, the pattern of cytokines and chemokines production was specific to the genera. We observed distinct clusters of response between the *Enterococcus* and the *Streptococcus*, and the strain *Pantoea EMP-364* induced a significant upregulation of most of the cytokines and chemokines we tested. Interestingly, we observed an significant production of MIF, up to 124,956 pg/mL (Figure S1, *EMS371*), induced by the *Streptococcus* strains. MIF overexpression has been shown to be linked to an increased level of IL-8, TNF-α, IL-6 and IL-1β in BAL fluid and sputum of asthmatic patients [46,47]. This proinflammatory cytokine also mediates the anti-inflammatory effect of glucocorticoids [47]. Herein, the high levels of MIF secreted in response to *Streptococcus* strains were not corelated to a significant production of IL-8, TNF-α, IL-6 and IL-1β by the BEAS-2B cells, under the experimental condition used. Previous observations showed that the secretome of a beneficial probiotic strain, *Lactobacillus rhamnosus* R0011, induced an important production of MIF by the HT-29 intestinal epithelial cells and attenuated proinflammatory mediators [48]. Moreover, MIF has been shown to induce MAPK activation within Type II alveolar epithelial cells, leading to enhanced cellular proliferation, potentially contributing to repair of damaged alveolus in response to infection [49]. Taken together, induction of MIF by the *Streptococcus* strains used in this study highlights a potential novel beneficial outcome of host–microbe interactions within the lung environment and warrants further investigation. We next thought to evaluate the differential gene expression of BEAS-2B, to refine our observations on the specificity of the response induced by the strains. The analysis of differential gene expression of BEAS-2B cells using genome-wide microarray showed that the *Streptococcus* and *Enterococcus* strains induced a differential gene expression profile. It is important to note here that no microarray data are available for one *Pantoea* (*EMP364*) and three *Streptococcus* (*EMS336*, *EMS353* and *EMS371*). These three strains of *Streptococcus* were shown to form a dense network in the culture medium during co-culture with epithelial cells. These networks might have stuck to the cell layer when recovering the supernatant, and we were never able to recover RNA following co-culture with these strains. This observation was specific to these three strains of *Streptococcus* only. *EMS321*, *EMS383* and *EMS3101* did not form such a network. It has been previously shown that oral *Streptococcus* possesses filamentous capacities that vary among species of the same genus [50]. Herein, we chose not to specify the species, for confidential reasons due to the partnership with an industrial company. Overall, the *Enterococcus* strains induced higher expression fold change for most of the genes involved in pro-inflammatory pathways. Genes such as *IL8* (maximum fold change = 14.50 for *EME343*), *CCL2* (maximum fold change = 9.67 for *EME343*), *ATF3* (maximum fold change = 7.89 for *EME343*), TNF-α Induced Protein 3 (*TNFAIP3*) (maximum fold change = 6.54 for *EME343*), Pentraxin 3 (*PTX3*) (maximum fold change = 6.24 for *EME343*), *CXCL2* (maximum fold change = 7.99 for *EME394*), *IL1A* (maximum fold change = 9.57 for *EME394*) and *CCL20* (maximum fold change = 11.19 for *EME394*) were the common top upregulated genes in cells incubated with the *Enterococcus* strains (Table 1). The enrichment analysis of the expression data showed an increased enrichment of molecular and cellular functions related to “inflammation”, “cell signaling”, and “cell-to-cell signaling and interactions” for the *Enterococcus* in regards to the *Streptococcus* (Figure 6). The analysis of the canonical pathway related to these clusters of molecular and cellular functions reinforced the differences observed between these two genera but also highlighted differences in regards to the strains. The gene sets were enriched in pathways such as glucocorticoid receptor signaling, IL-6 signaling, role of IL-17F in allergic inflammatory airway diseases, IL-17 signaling, TNFR1 and 2 signaling, IL-10 signaling and JAK/STAT signaling (Figure 7). These pathways are downstream pathways of cytokines signal transduction and play an important role in the immune response of the host [51,52,53]. Altogether, these differences suggest that, in response to the bacterial stimuli, the epithelial cells might respond specifically by recruiting and stimulating different immune cell populations, depending on their phylogeny.

Newborn lungs, as the immune system, are fully functioning at birth but still developing, and several factors can influence normal morphogenesis. In humans, lung formation starts during the embryonic phase, and the alveolarization finishes at early adult age. Herein, we observed the differential expression of some genes related to the regulation of the postnatal lung morphogenesis such as *EGR1* and *VEGFA* (Table 1). Vascular endothelial growth factor (VEGF) signaling has been shown to play a role in early lung morphogenesis, and BAL VEGF levels may be used to identify preterm infants with a risk of developing BPD [54,55]. The growth factor VEGF was detectable and measurable with the multiplex analysis, but the high variability between replicates did not allow the assessment of the extent of VEGF production (data not shown). We found a high number of genes significantly enriched for the glucocorticoid receptor-signaling pathway. Glucocorticoid receptors, once activated in developing lung, induce a series of morphological alteration in the pulmonary architecture and stimulate lung maturation [56]. Moreover, glucocorticoid treatments were shown to be effective in infant at risk to develop respiratory diseases and are commonly used in women at risk of preterm birth in order to help mature fetal lungs [56,57,58]. We also noted differential production of basic-FGF, C-GSF, GM-CSF and IL-1b that have all been shown to be involved in lung morphogenesis. Similar to their potential impact on the pulmonary immune maturation, the strains studied in this project may have a differential impact on the morphological and functional maturation of the airways. 

To our knowledge, this is the first study to establish the impact of bacterial strains, isolated from the buccal cavity during the first week of life of two babies, on human bronchial epithelial cells in vitro. Although it gives new insight into the impact of different bacterial genera of the oral cavity to modulate lung epithelial cells responses, our study has some limitations. First, the bacteria studied in this project originated from two donors. Even though the strains isolated in our study are in accordance with previous reports on the composition of the BC microbiota, sampling should be performed on a higher number of babies, in order to be more representative of the early-life buccal microbiota. A metagenomic study would give a larger view of the total microbiota and allow us to assess the impact of the sampling, storage and transport methods on the amount and diversity of the bacterial strains recovered. Secondly, we have used BEAS-2B cells as model of bronchial epithelial cells. While providing several advantages, such as low variability and easy handling, BEAS-2B cells represented a simplification of the bronchial epithelium. Indeed, the monolayered undifferentiated cell culture implies a lack of the different cell types (basal, ciliated, goblet and club cells), which were shown to exert specific roles in the epithelial physiology of the airways. For further analysis, it would be of interest to use primary airway epithelial cells and/or a reconstituted airway epithelia to better understand the effects of these strains on immune homeostasis and especially in a pro-inflammatory context. Finally, it will be important to test these strains in vivo, to investigate their capacity to stimulate the lung immunity and to provide an advantage against the development of chronic or acute respiratory diseases.

## Figures and Tables

**Table 1:** Top 10 differentially upregulated genes identified by genome-wide human expression microarrays. * Genes were considered significantly differentially expressed when (a) a t-test *p-*value was less than 0.05 and (b) a cutoff in transcript abundance reached at least 1.5-fold change. The 10 most upregulated genes for each strain are displayed in the table.

*EME343*		*EME394*		*EME126*		*EME141*		*EMS321*		*EMS383*		*EMS3101*	
Gene	Fold Change	Gene	Fold Change	Gene	Fold Change	Gene	Fold Change	Gene	Fold Change	Gene	Fold Change	Gene	Fold Change
*IL8*	14.502	*IL8*	12.219	*IL8*	8.043	*CCL2*	4.718	*ATF3*	5.989	*CCL2*	6.741	*SNORD3B-1*	4.091
*CCL2*	9.667	*CCL20*	11.190	*CCL2*	6.391	*GADD45A*	3.706	*IL8*	5.036	*C1QTNF9*	6.711	*HIST2H2AA4*	3.981
*ATF3*	7.888	*IL1A*	9.573	*TNFAIP3*	4.777	*PTX3*	3.620	*CCL2*	3.968	*SULT1B1*	5.156	*CCL2*	3.108
*TNFAIP3*	6.536	*CXCL2*	7.994	*CLDN1*	3.609	*DDIT3*	3.497	*EGR1*	3.734	*CLDN1*	4.469	*SLC5A7*	3.086
*PTX3*	6.242	*CCL2*	7.351	*IER3*	3.527	*CLDN1*	3.257	*STC2*	3.732	*CHAC1*	4.310	*HIST1H2BI*	3.074
*CDKN1A*	6.132	*PTX3*	5.996	*NDRG1*	3.296	*STC2*	3.160	*PTX3*	3.420	*ATF3*	4.124	*HIST1H2AC*	2.910
*IL1A*	6.088	*EGR1*	4.945	*CXCL1*	3.220	*CXCL2*	3.052	*DDIT*3	3.307	*ASNS*	4.020	*HIST1H2BC*	2.848
*SLC7A11*	5.094	*STC2*	4.444	*PTX3*	2.892	*MAFF*	2.901	*CHAC1*	3.166	*TRIB3*	3.930	*HIST2H4B*	2.759
*EGR1*	4.936	*ATF3*	4.391	*CSN1S1*	2.471	*PPP1R15A*	2.601	*CLDN1*	3.116	*STC2*	3.792	*HIST1H4L*	2.676
*CXCL2*	4.823	*CHAC1*	4.344	*CEBPB*	2.432	*EGR1*	2.573	*VEGFA*	2.963	*SLC7A11*	3.502	*HIST4H4*	2.593

## Supplementary Materials

The following are available online at https://www.mdpi.com/2076-2607/8/8/1094/s1. Figure S1: Cytokines and chemokines profiling of the BEAS-2B cells in response to bacterial incubation. Figure S2: Cytotoxicity induced by early life oral bacteria on BEAS-2B cells after 6 h treatment. Table S1: Fold changes for each strain and for each gene presented in Table 1.
