# Supplementary material for: Oral Primo-Colonizing Bacteria Modulate Inflammation and Gene Expression in Bronchial Epithelial Cells
Source: Microorganisms. 2020 Jul 22;8(8):1094. doi: 10.3390/microorganisms8081094 (PMC7464694; doi:10.3390/microorganisms8081094)
Supplement: Supplementary file 1 [file microorganisms-08-01094-s001.zip › Supplemental Figures Mathieu Elliot et al., 2020/Supplemental Figures.pptx]

## Slide 1
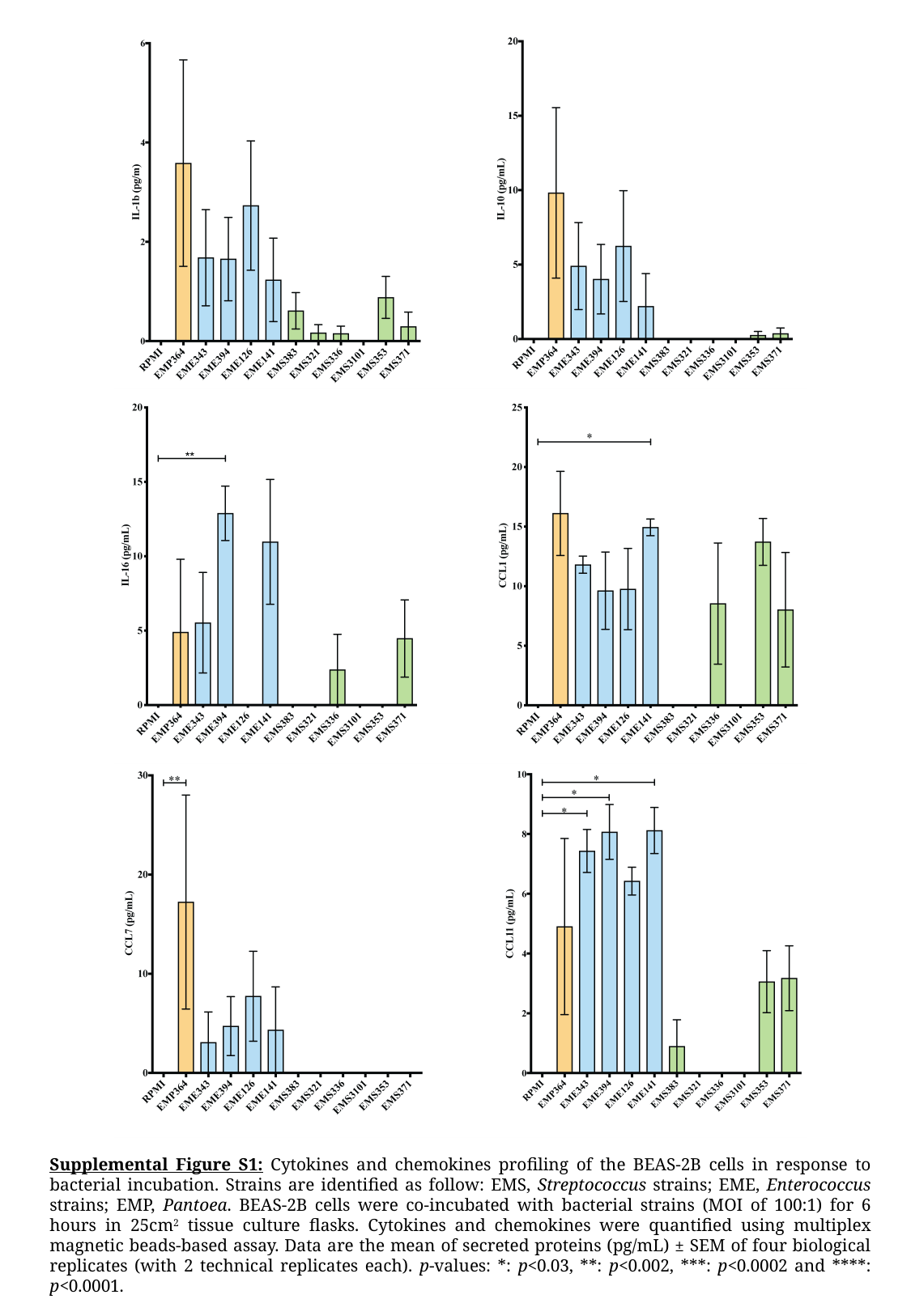

Supplemental Figure S1: Cytokines and chemokines profiling of the BEAS-2B cells in response to bacterial incubation. Strains are identified as follow: EMS, Streptococcus strains; EME, Enterococcus strains; EMP, Pantoea. BEAS-2B cells were co-incubated with bacterial strains (MOI of 100:1) for 6 hours in 25cm2 tissue culture flasks. Cytokines and chemokines were quantified using multiplex magnetic beads-based assay. Data are the mean of secreted proteins (pg/mL) ± SEM of four biological replicates (with 2 technical replicates each). p-values: *: p<0.03, **: p<0.002, ***: p<0.0002 and ****: p<0.0001.

## Slide 2
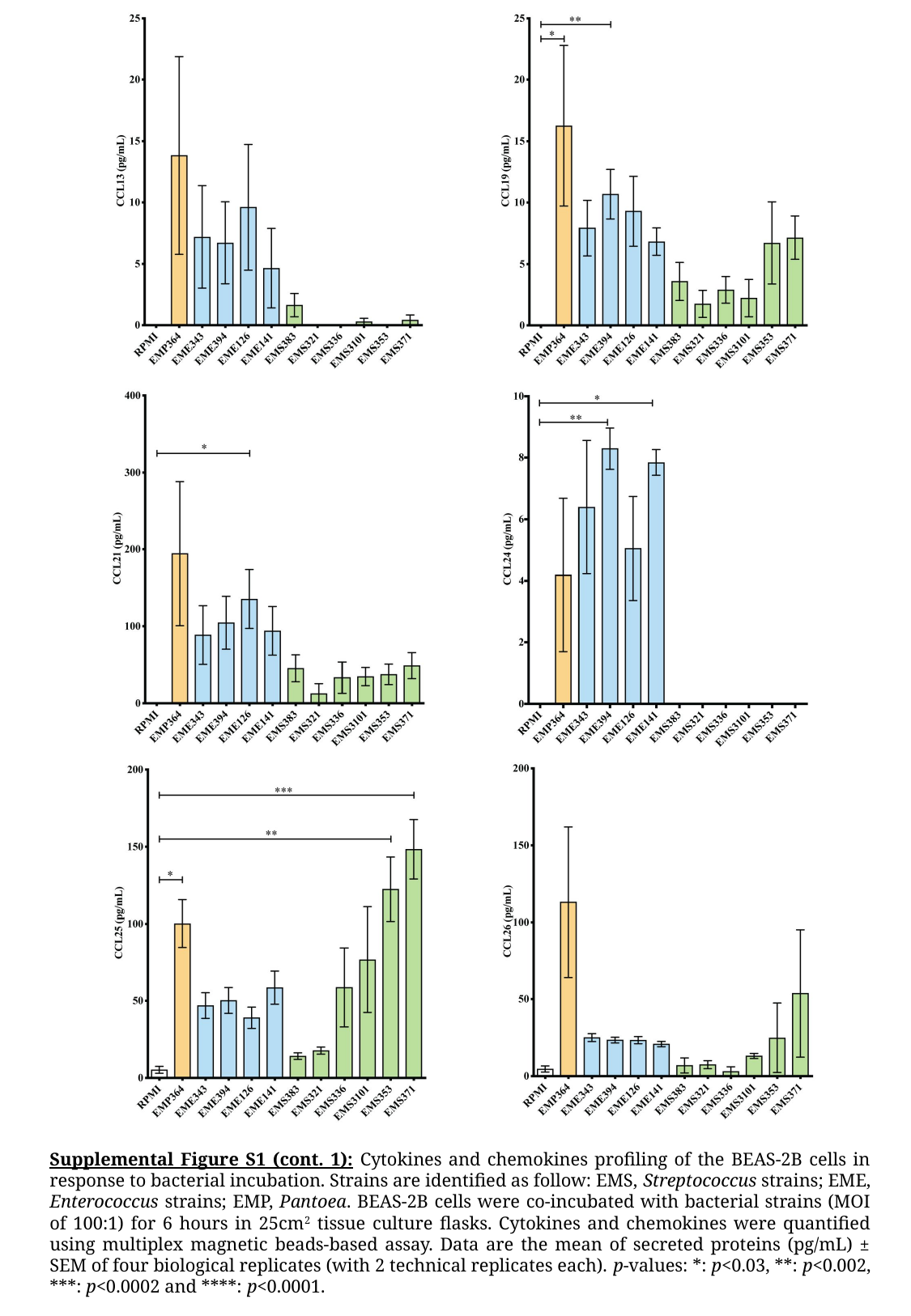

Supplemental Figure S1 (cont. 1): Cytokines and chemokines profiling of the BEAS-2B cells in response to bacterial incubation. Strains are identified as follow: EMS, Streptococcus strains; EME, Enterococcus strains; EMP, Pantoea. BEAS-2B cells were co-incubated with bacterial strains (MOI of 100:1) for 6 hours in 25cm2 tissue culture flasks. Cytokines and chemokines were quantified using multiplex magnetic beads-based assay. Data are the mean of secreted proteins (pg/mL) ± SEM of four biological replicates (with 2 technical replicates each). p-values: *: p<0.03, **: p<0.002, ***: p<0.0002 and ****: p<0.0001.

## Slide 3
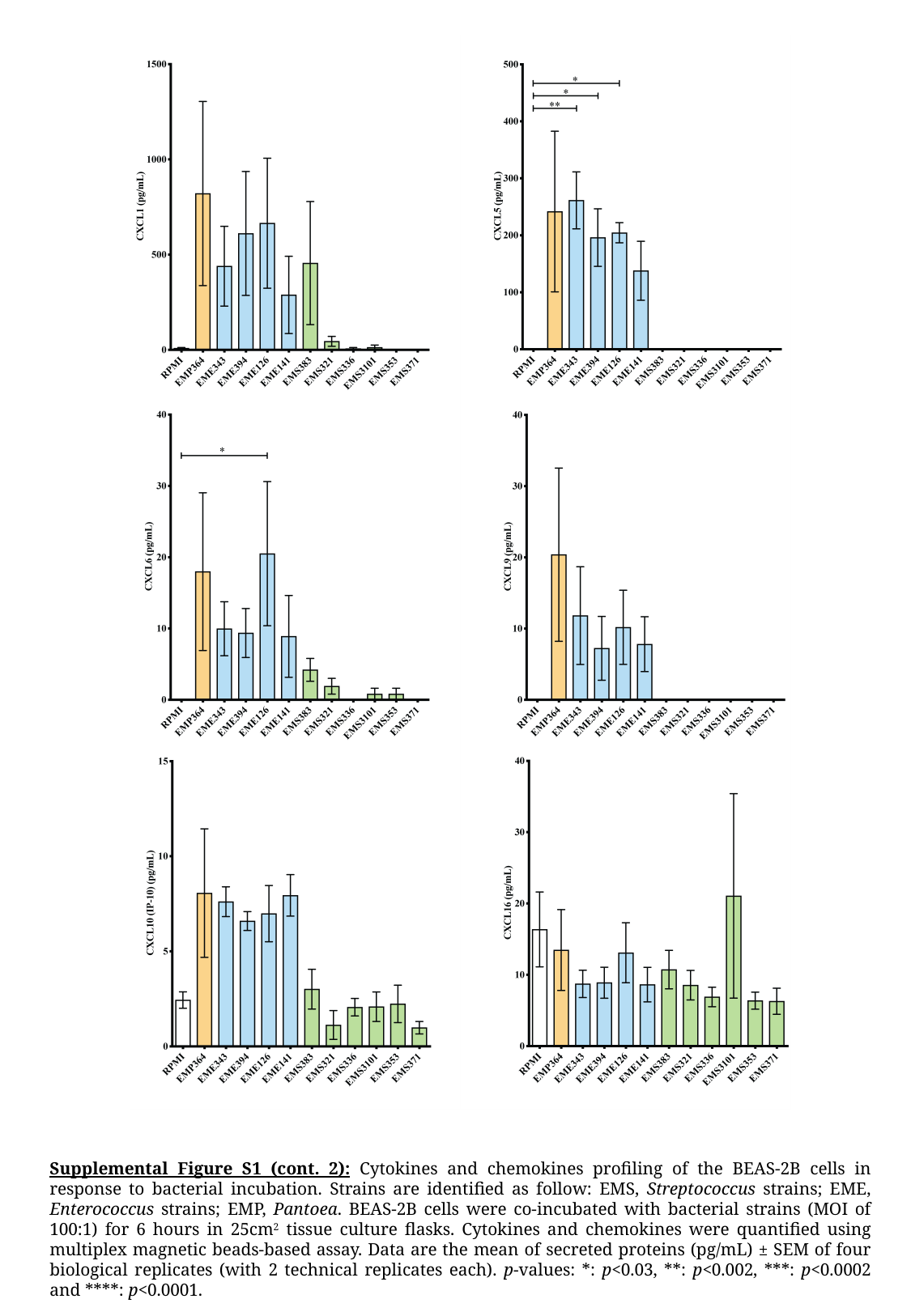

Supplemental Figure S1 (cont. 2): Cytokines and chemokines profiling of the BEAS-2B cells in response to bacterial incubation. Strains are identified as follow: EMS, Streptococcus strains; EME, Enterococcus strains; EMP, Pantoea. BEAS-2B cells were co-incubated with bacterial strains (MOI of 100:1) for 6 hours in 25cm2 tissue culture flasks. Cytokines and chemokines were quantified using multiplex magnetic beads-based assay. Data are the mean of secreted proteins (pg/mL) ± SEM of four biological replicates (with 2 technical replicates each). p-values: *: p<0.03, **: p<0.002, ***: p<0.0002 and ****: p<0.0001.

## Slide 4
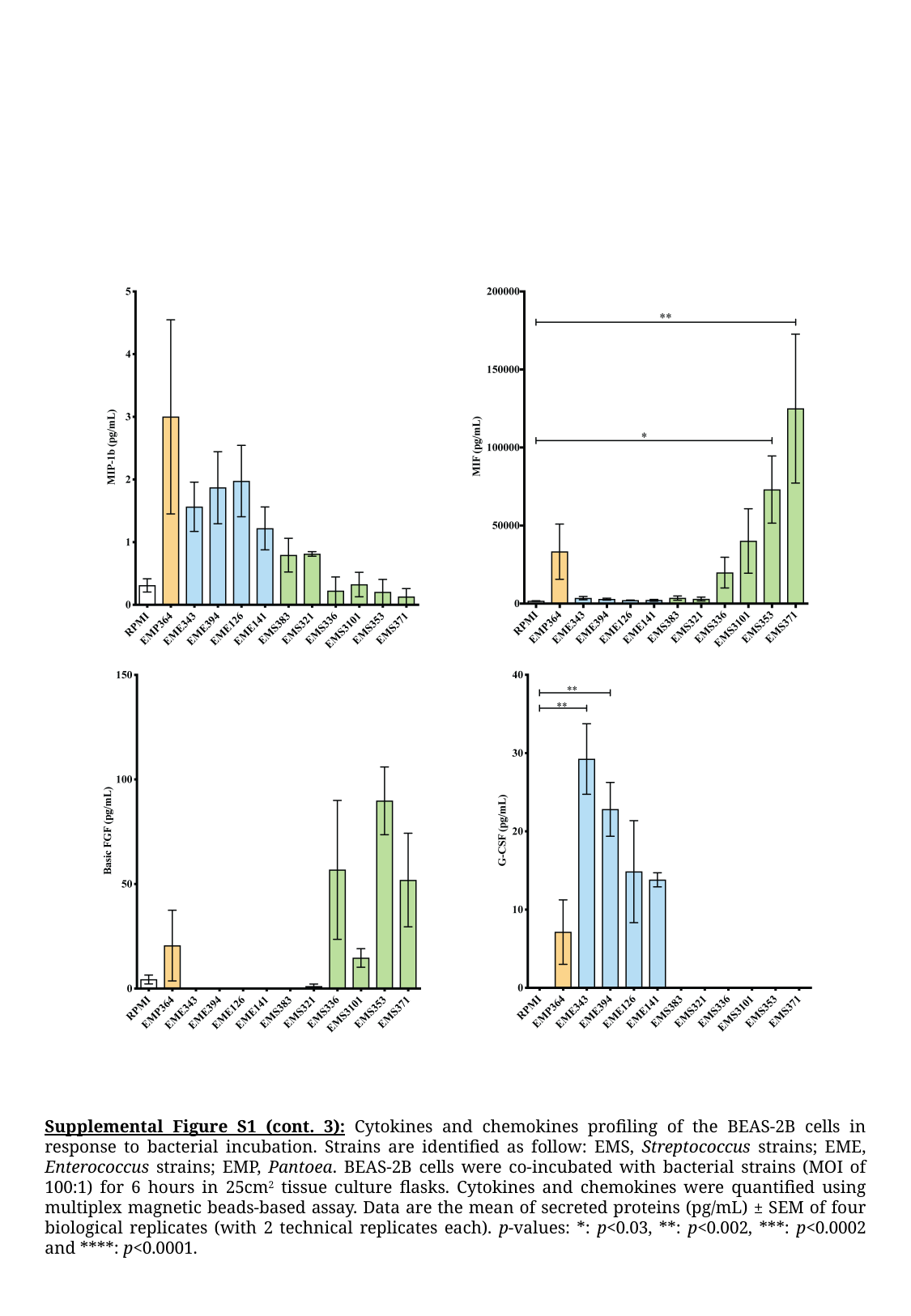

Supplemental Figure S1 (cont. 3): Cytokines and chemokines profiling of the BEAS-2B cells in response to bacterial incubation. Strains are identified as follow: EMS, Streptococcus strains; EME, Enterococcus strains; EMP, Pantoea. BEAS-2B cells were co-incubated with bacterial strains (MOI of 100:1) for 6 hours in 25cm2 tissue culture flasks. Cytokines and chemokines were quantified using multiplex magnetic beads-based assay. Data are the mean of secreted proteins (pg/mL) ± SEM of four biological replicates (with 2 technical replicates each). p-values: *: p<0.03, **: p<0.002, ***: p<0.0002 and ****: p<0.0001.

## Slide 5
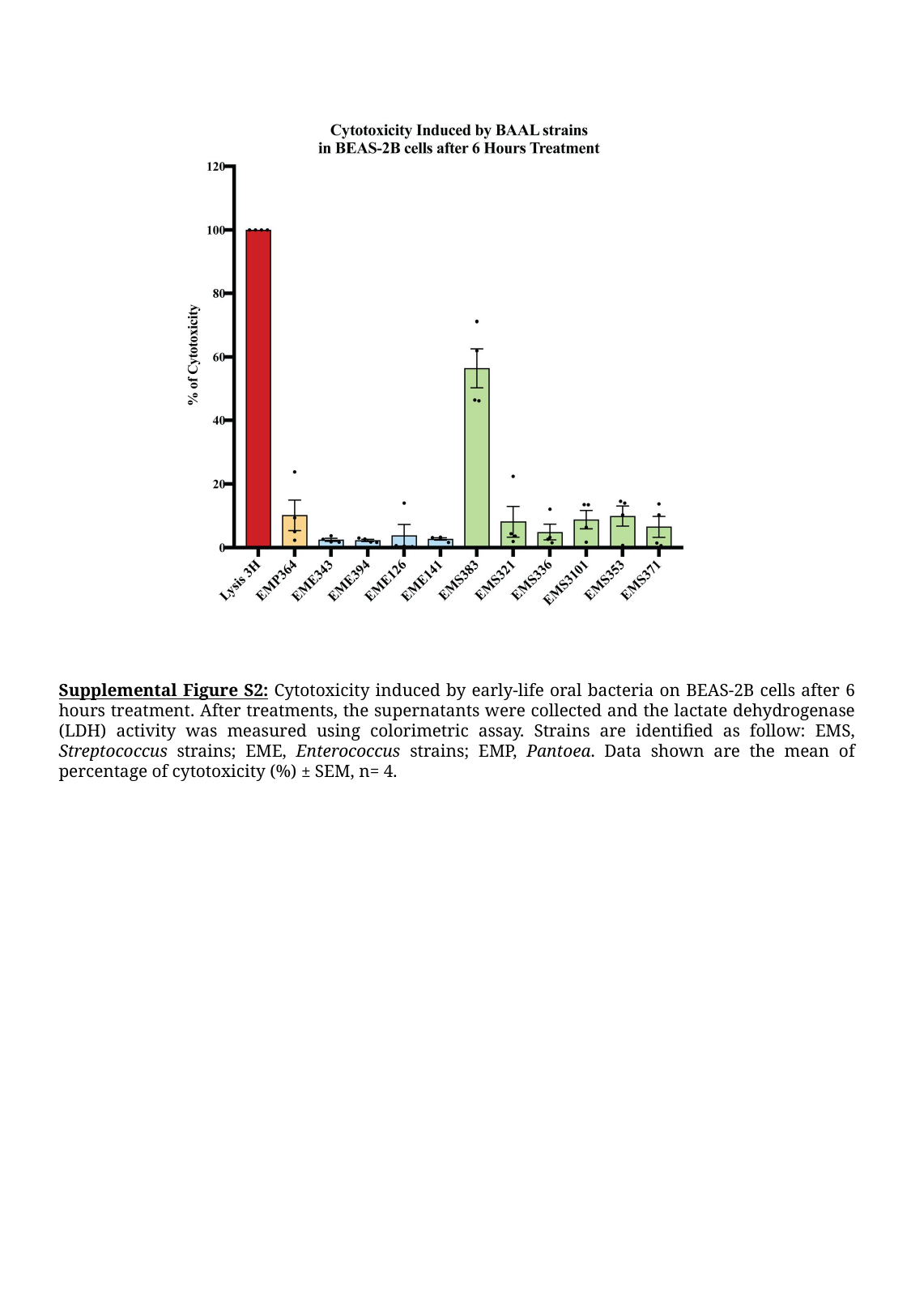

Supplemental Figure S2: Cytotoxicity induced by early-life oral bacteria on BEAS-2B cells after 6 hours treatment. After treatments, the supernatants were collected and the lactate dehydrogenase (LDH) activity was measured using colorimetric assay. Strains are identified as follow: EMS, Streptococcus strains; EME, Enterococcus strains; EMP, Pantoea. Data shown are the mean of percentage of cytotoxicity (%) ± SEM, n= 4.

## Slide 6
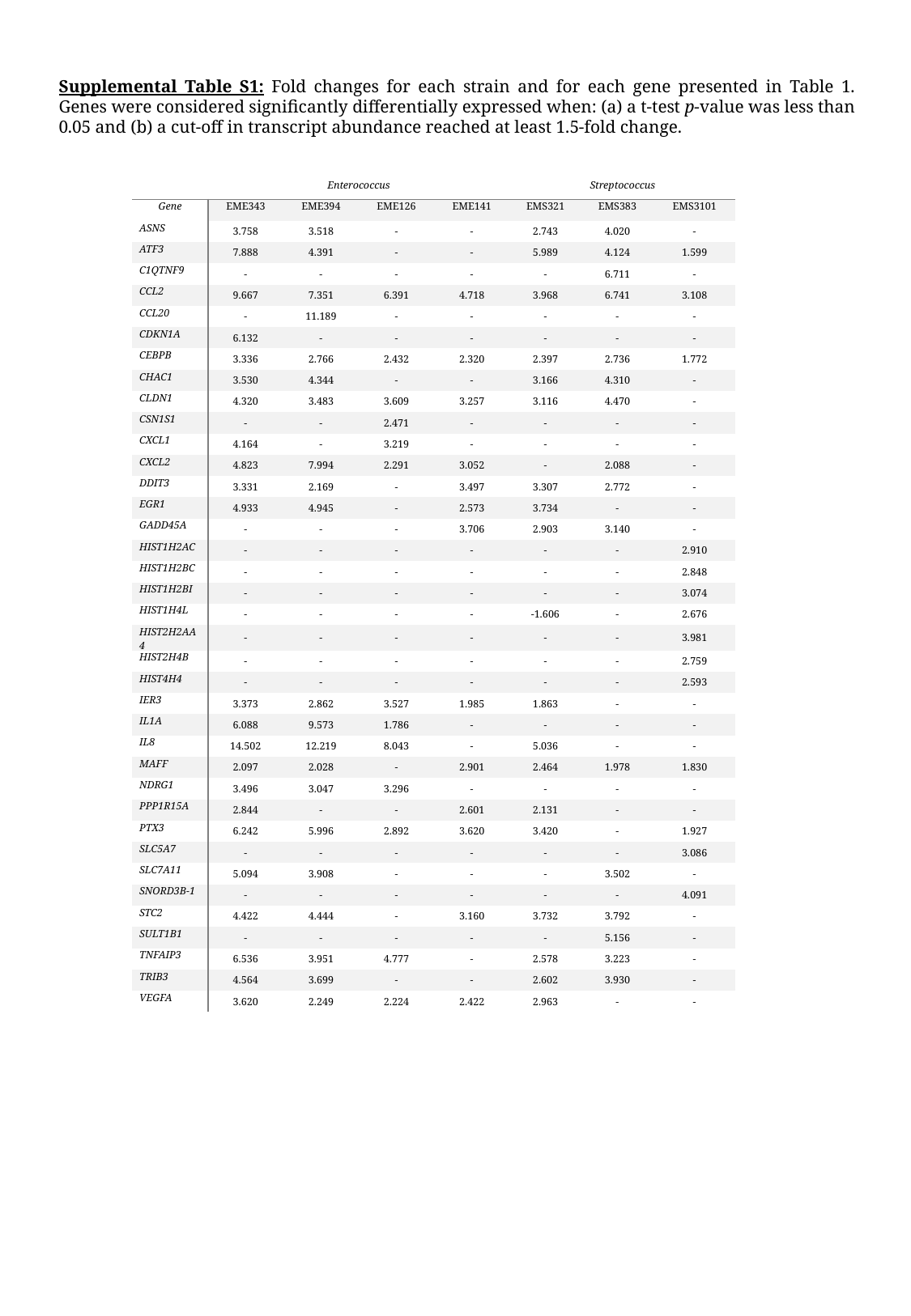

Supplemental Table S1: Fold changes for each strain and for each gene presented in Table 1. Genes were considered significantly differentially expressed when: (a) a t-test p-value was less than 0.05 and (b) a cut-off in transcript abundance reached at least 1.5-fold change.
| | Enterococcus | | | | Streptococcus | | |
| --- | --- | --- | --- | --- | --- | --- | --- |
| Gene | EME343 | EME394 | EME126 | EME141 | EMS321 | EMS383 | EMS3101 |
| ASNS | 3.758 | 3.518 | - | - | 2.743 | 4.020 | - |
| ATF3 | 7.888 | 4.391 | - | - | 5.989 | 4.124 | 1.599 |
| C1QTNF9 | - | - | - | - | - | 6.711 | - |
| CCL2 | 9.667 | 7.351 | 6.391 | 4.718 | 3.968 | 6.741 | 3.108 |
| CCL20 | - | 11.189 | - | - | - | - | - |
| CDKN1A | 6.132 | - | - | - | - | - | - |
| CEBPB | 3.336 | 2.766 | 2.432 | 2.320 | 2.397 | 2.736 | 1.772 |
| CHAC1 | 3.530 | 4.344 | - | - | 3.166 | 4.310 | - |
| CLDN1 | 4.320 | 3.483 | 3.609 | 3.257 | 3.116 | 4.470 | - |
| CSN1S1 | - | - | 2.471 | - | - | - | - |
| CXCL1 | 4.164 | - | 3.219 | - | - | - | - |
| CXCL2 | 4.823 | 7.994 | 2.291 | 3.052 | - | 2.088 | - |
| DDIT3 | 3.331 | 2.169 | - | 3.497 | 3.307 | 2.772 | - |
| EGR1 | 4.933 | 4.945 | - | 2.573 | 3.734 | - | - |
| GADD45A | - | - | - | 3.706 | 2.903 | 3.140 | - |
| HIST1H2AC | - | - | - | - | - | - | 2.910 |
| HIST1H2BC | - | - | - | - | - | - | 2.848 |
| HIST1H2BI | - | - | - | - | - | - | 3.074 |
| HIST1H4L | - | - | - | - | -1.606 | - | 2.676 |
| HIST2H2AA4 | - | - | - | - | - | - | 3.981 |
| HIST2H4B | - | - | - | - | - | - | 2.759 |
| HIST4H4 | - | - | - | - | - | - | 2.593 |
| IER3 | 3.373 | 2.862 | 3.527 | 1.985 | 1.863 | - | - |
| IL1A | 6.088 | 9.573 | 1.786 | - | - | - | - |
| IL8 | 14.502 | 12.219 | 8.043 | - | 5.036 | - | - |
| MAFF | 2.097 | 2.028 | - | 2.901 | 2.464 | 1.978 | 1.830 |
| NDRG1 | 3.496 | 3.047 | 3.296 | - | - | - | - |
| PPP1R15A | 2.844 | - | - | 2.601 | 2.131 | - | - |
| PTX3 | 6.242 | 5.996 | 2.892 | 3.620 | 3.420 | - | 1.927 |
| SLC5A7 | - | - | - | - | - | - | 3.086 |
| SLC7A11 | 5.094 | 3.908 | - | - | - | 3.502 | - |
| SNORD3B-1 | - | - | - | - | - | - | 4.091 |
| STC2 | 4.422 | 4.444 | - | 3.160 | 3.732 | 3.792 | - |
| SULT1B1 | - | - | - | - | - | 5.156 | - |
| TNFAIP3 | 6.536 | 3.951 | 4.777 | - | 2.578 | 3.223 | - |
| TRIB3 | 4.564 | 3.699 | - | - | 2.602 | 3.930 | - |
| VEGFA | 3.620 | 2.249 | 2.224 | 2.422 | 2.963 | - | - |
