# Supplementary figures and images for: Oral Primo-Colonizing Bacteria Modulate Inflammation and Gene Expression in Bronchial Epithelial Cells
Source: Microorganisms. 2020 Jul 22;8(8):1094. doi: 10.3390/microorganisms8081094 (PMC7464694; doi:10.3390/microorganisms8081094)

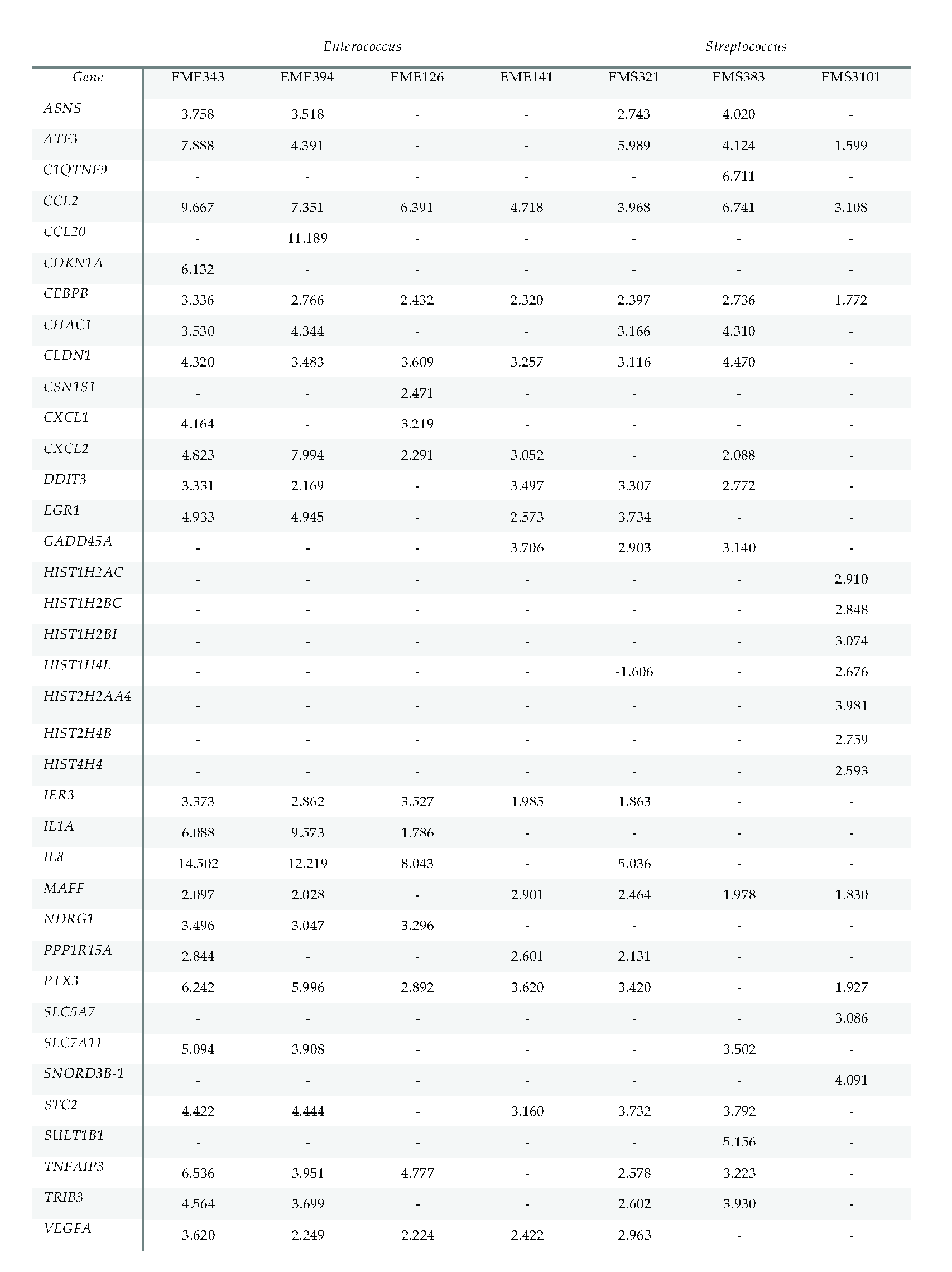

Supplement: Supplementary file 1 [file microorganisms-08-01094-s001.zip › Supplemental Figures Mathieu Elliot et al., 2020/Supplemental Table S1.tif]

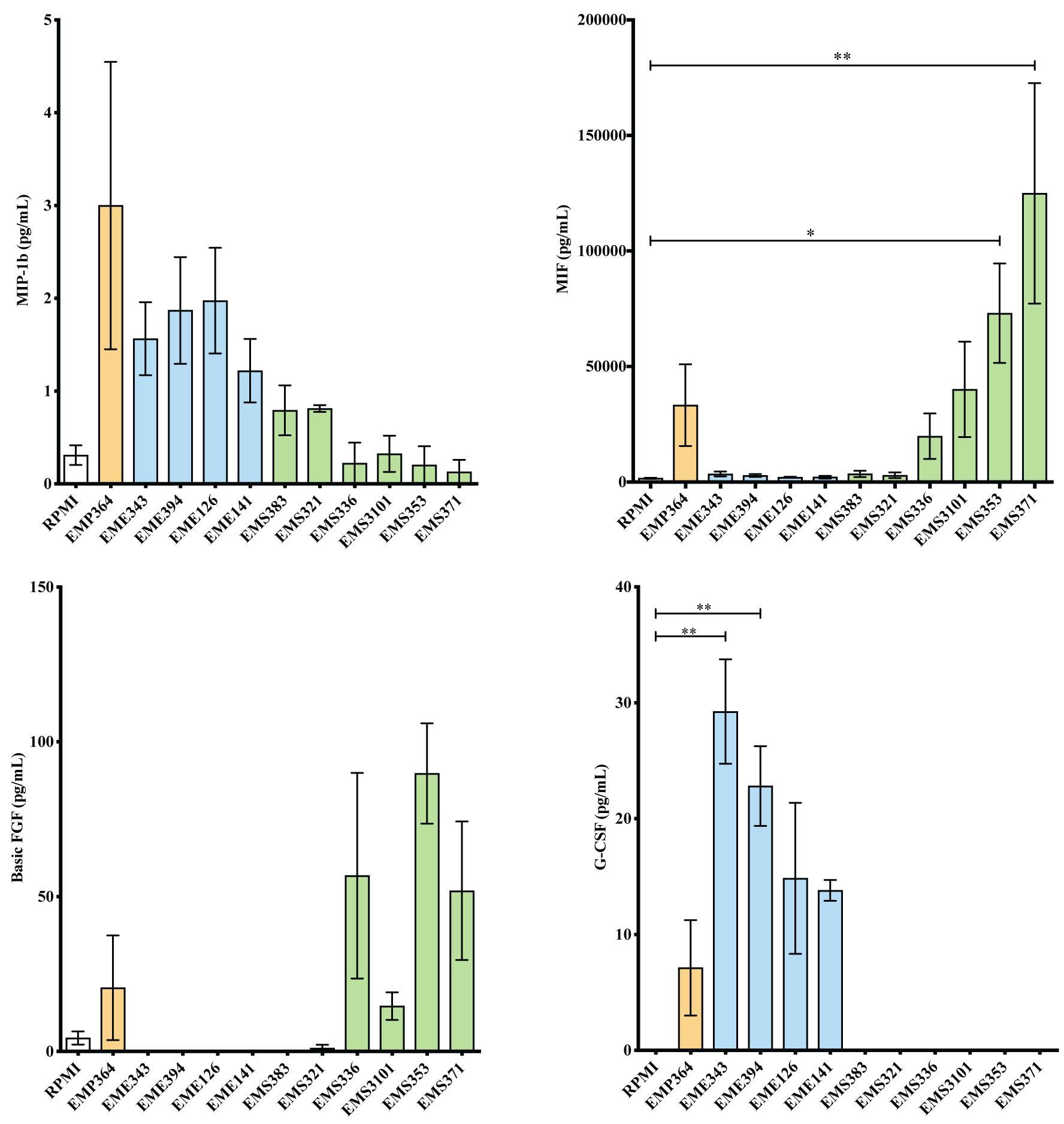

Supplement: Supplementary file 1 [file microorganisms-08-01094-s001.zip › Supplemental Figures Mathieu Elliot et al., 2020/Figure S1 (cont. 3).tif]

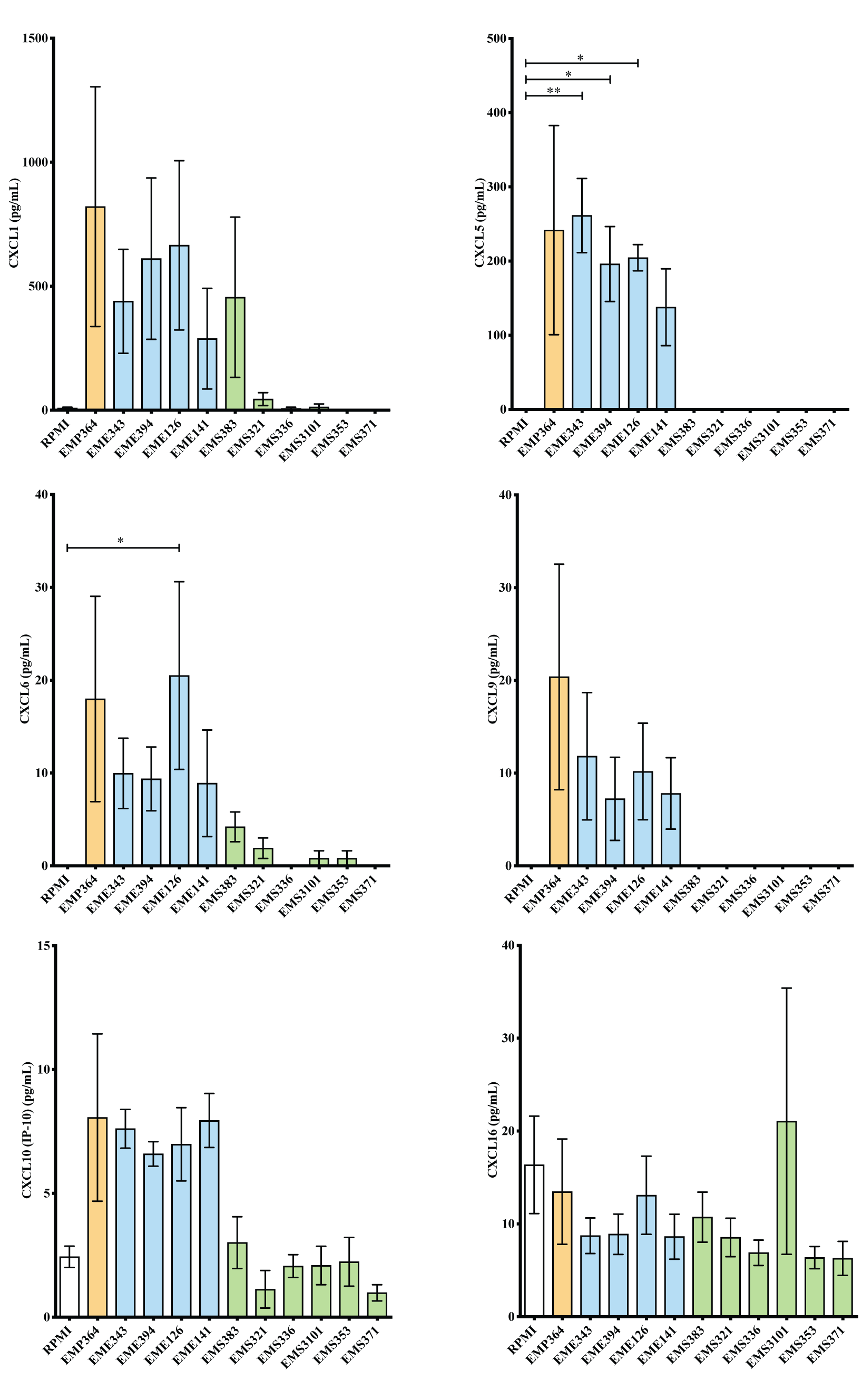

Supplement: Supplementary file 1 [file microorganisms-08-01094-s001.zip › Supplemental Figures Mathieu Elliot et al., 2020/Figure S1 (cont. 2).tif]

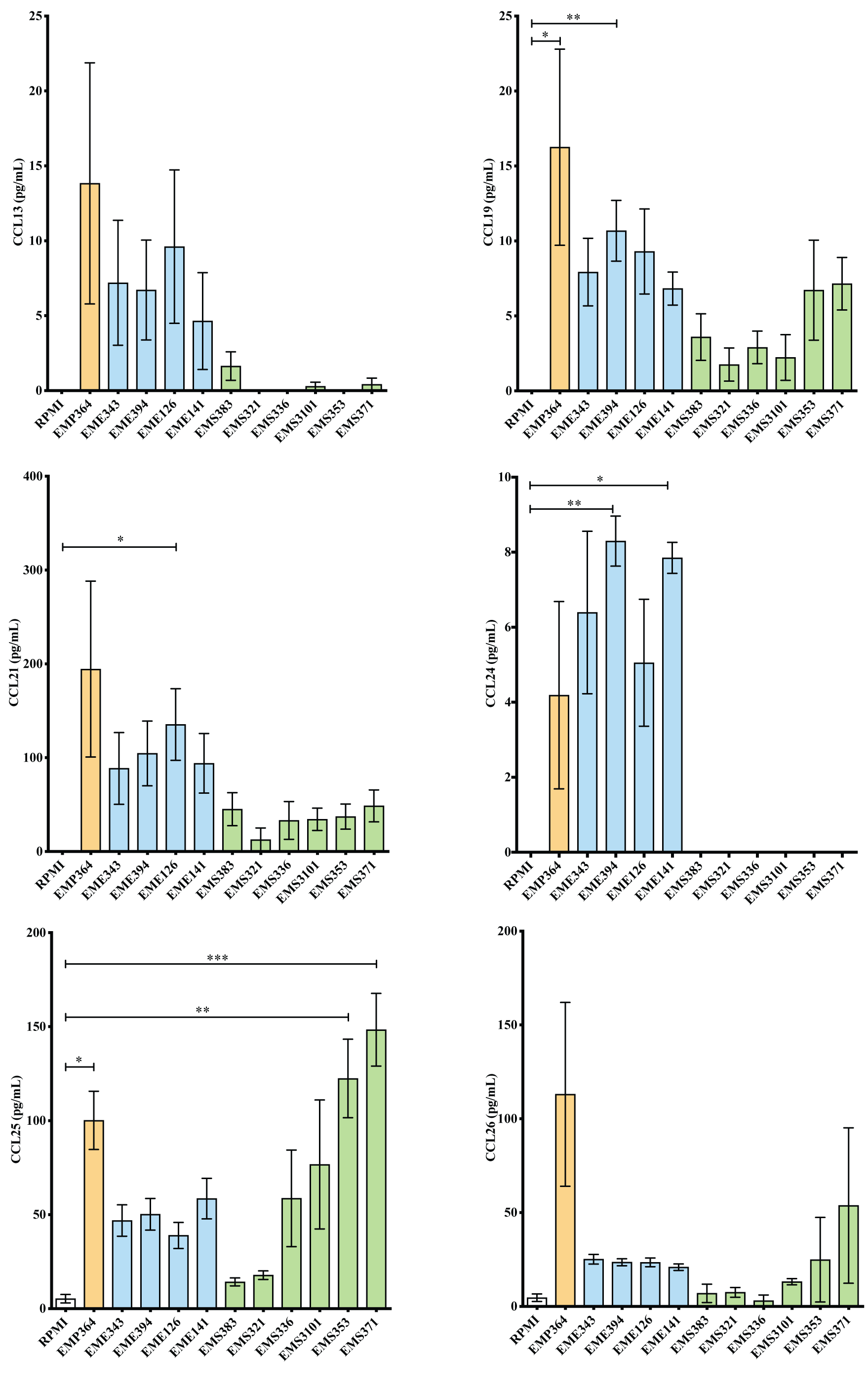

Supplement: Supplementary file 1 [file microorganisms-08-01094-s001.zip › Supplemental Figures Mathieu Elliot et al., 2020/Figure S1 (cont. 1).tif]

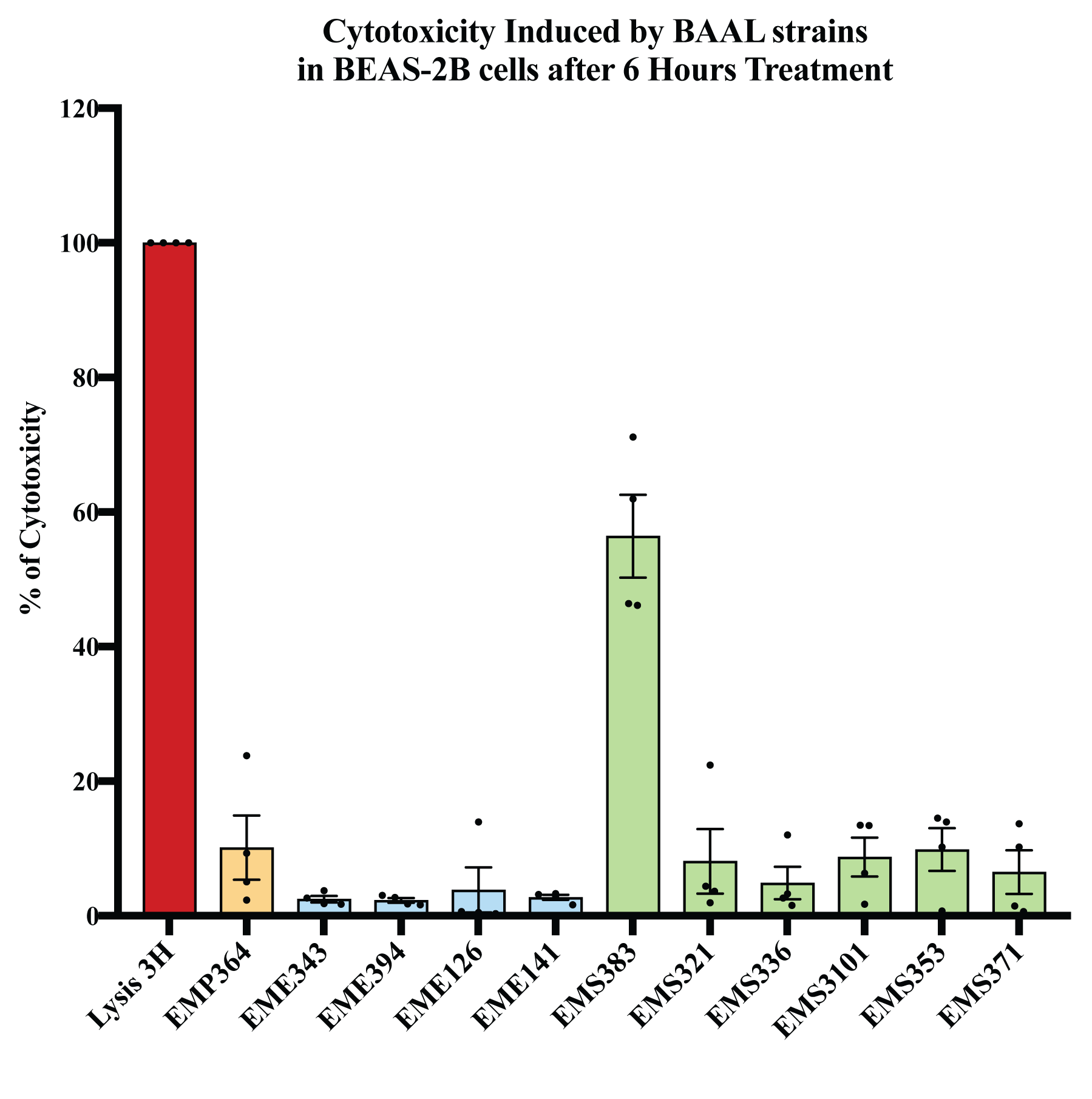

Supplement: Supplementary file 1 [file microorganisms-08-01094-s001.zip › Supplemental Figures Mathieu Elliot et al., 2020/Figure S2.tif]

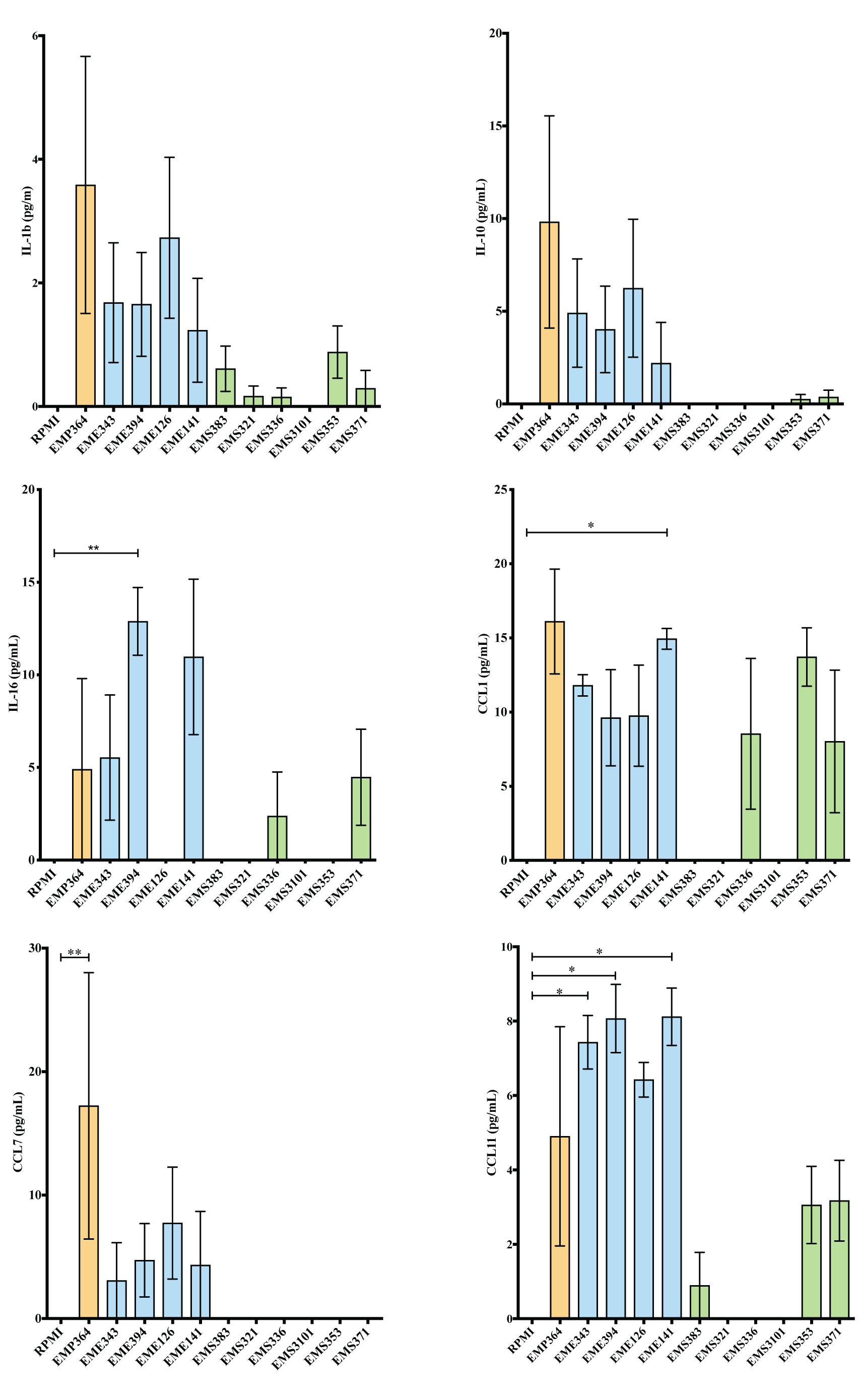

Supplement: Supplementary file 1 [file microorganisms-08-01094-s001.zip › Supplemental Figures Mathieu Elliot et al., 2020/Figure S1.tif]
